# Sub-Nanoscale Surface Ruggedness Provides a Water-Tight Seal for Exposed Regions in Soluble Protein Structure

**DOI:** 10.1371/journal.pone.0012844

**Published:** 2010-09-17

**Authors:** Erica Schulz, Marisa Frechero, Gustavo Appignanesi, Ariel Fernández

**Affiliations:** 1 Sección Fisicoquímica, Instituto de Química del Sur, Universidad Nacional del Sur, Consejo Nacional de Investigaciones Científicas y Técnicas and Departamento de Química, Universidad Nacional del Sur, Bahía Blanca, Argentina; 2 Department of Bioengineering, Rice University, Houston, Texas, United States of America; 3 Department of Computer Science, The University of Chicago, Chicago, Illinois, United States of America; Indiana University, United States of America

## Abstract

Soluble proteins must maintain backbone hydrogen bonds (BHBs) water-tight to ensure structural integrity. This protection is often achieved by burying the BHBs or wrapping them through intermolecular associations. On the other hand, water has low coordination resilience, with loss of hydrogen-bonding partnerships carrying significant thermodynamic cost. Thus, a core problem in structural biology is whether natural design actually exploits the water coordination stiffness to seal the backbone in regions that are exposed to the solvent. This work explores the molecular design features that make this type of seal operative, focusing on the side-chain arrangements that shield the protein backbone. We show that an efficient sealing is achieved by adapting the sub-nanoscale surface topography to the stringency of water coordination: *an exposed BHB may be kept dry if the local concave curvature is small enough to impede formation of the coordination shell of a penetrating water molecule*. Examination of an exhaustive database of uncomplexed proteins reveals that exposed BHBs invariably occur within such sub-nanoscale cavities in native folds, while this level of local ruggedness is absent in other regions. By contrast, BHB exposure in misfolded proteins occurs with larger local curvature promoting backbone hydration and consequently, structure disruption. These findings unravel physical constraints fitting a spatially dependent least-action for water coordination, introduce a molecular design concept, and herald the advent of water-tight peptide-based materials with sufficient backbone exposure to remain flexible.

## Introduction

The loss of hydrogen-bonded partners implies a significant thermodynamic cost for a water molecule [1]–[4]. Here we show that this low coordination resilience, whereby water tends to maintain its tetrahedral lattice of hydrogen-bonding partnerships, is instrumental in providing a seal for soluble proteins. By stabilizing the unfolded state, backbone hydration may dismantle a protein fold if the latter over-exposes the backbone [5], [6]. However, and this is the point of this work, water tightness may be achieved even in structured regions with backbone exposure to the solvent: The backbone-water hydrogen bond may not materialize if its formation requires that the cavity-penetrating water molecule relinquish a significant fraction of its coordination possibilities. In this sense, the low resilience of water coordination [1], [2] imposes physical constraints on the natural design of stable interfaces for soluble structures, as shown in this work.

To maintain structural integrity, soluble proteins often protect backbone amide-carbonyl hydrogen bonds (BHBs, Methods) by “wrapping” them with surrounding side-chain nonpolar groups [4], [8]. Here we show that an alternative sealing occurs for under-wrapped structures through a fine tuning of surface ruggedness. Ruggedness refers throughout this work to the presence of sub-nanoscale cavities disrupting surface smoothness. Rugged protein structures maintain their integrity by promoting dehydration of exposed backbone groups aptly named *dehydrons* [4], [6]. This dehydration propensity has been inferred previously from first principles and validated using structural data [4], but no molecular-level rationale has been provided linking dehydration to local surface curvature in monomeric free proteins, as done in this work.

An exhaustive analysis of uncomplexed proteins reveals that under-wrapped backbone regions present cavities fine tuned (∼1 to 3.5 Å-curvature radius) to exclude water by curtailing its hydrogen-bonding capabilities. This exclusion may be relaxed if water is capable of forming more than one hydrogen bond with the protein, as in water-mediated intramolecular interactions [4]. Since a water molecule cannot accommodate its coordination domain inside a sub-nanoscale cavity, water effectively distances itself from exposed BHBs to a larger extent than from well protected bonds. This level of sub-nanoscale ruggedness is absent, and in fact unnecessary, in well-wrapped proteins. By contrast, as shown below, in the paradigmatic example of neurotoxins [9], the most under-wrapped proteins [4], sub-nanoscale topography ensures structural integrity. On the other hand, misfolded proteins or structural decoys [10] do not fulfill the same geometric constraints and hence are unable to prevent hydration of exposed BHBs. In contrast with previous studies on protein hydration [3], [11], [12], the focus here is the relationship between structural integrity and de-wetting patterns that exploit the low coordination resilience of interfacial water.

## Results and Discussion

**Figure 1:**
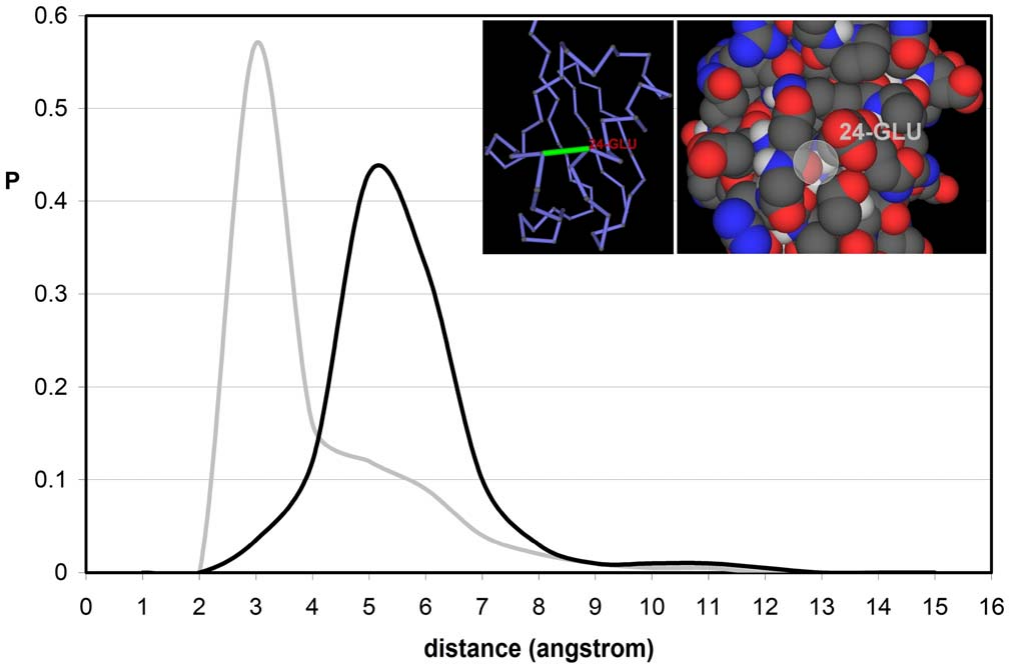
Distances from interfacial water to exposed and buried backbone hydrogen bonds in soluble proteins. Probability distribution of distances from backbone hydrogen bonds (BHBs, grey line) and exposed backbone hydrogen bonds (EBHBs, black line) to the closest surrounding water molecule. The distributions were obtained from the equilibrium values of the parameter d_min_ for 377,116 BHBs and 97,120 EBHBs for an exhaustive dataset of 2661 free monomeric proteins reported in PDB (Table S1). The inset displays in virtual bond and space filling representation the EBHB involving residues Asp52-Glu29 of human ubiquitin (PDB.1UBI) with osculating sphere radius θ = 1.7Å.

The exposed backbone hydrogen bonds (EBHBs) represent a class of structural vulnerability whereby the bond is insufficiently wrapped or shielded from solvent by side-chain nonpolar groups [4], [6]. These singularities may be identified in soluble structures by determining the number of side-chain nonpolar groups –the so-called wrappers- contained within the bond local environment (Methods). These backbone bonds cannot afford hydration as this would make the structure unsustainable, yet they are *de facto* exposed to the solvent. This introduces a conundrum as solvent exposure can only be reconciled with structural stability if we assume that water cannot penetrate available interfacial space. This is indeed the case as shown in Fig. 1, obtained from a nonredundant exhaustive set of 2661 PDB-reported structures of monomeric uncomplexed proteins, each equilibrated using molecular dynamics within an NPT ensemble (Methods). The computations start with the PDB-reported structure embedded in a pre-equilibrated cell of water molecules [13], [14].

To determine the location of the closest water molecule around a BHB, we compute the minimum distance between the oxygens of carbonyl and water. This convention is adopted for computational simplicity, given the proximity of the water oxygen to the baricenter of the molecule. The results obtained when adopting the amide nitrogen *in lieu* of the carbonyl oxygen are statistically indistinguishable. Fig. 1 displays the distribution of distances of the closest water molecules for all BHBs for the exhaustive structural database discriminating between well wrapped BHBs (grey line) and EBHBs (black line). *Strikingly, water gets closer to a well-wrapped BHB, with most probable distance ∼3Å, than to an exposed bond, where the distance distribution peaks at ∼5Å*.

The most probable distance for well-wrapped BHBs is consistent with the typical arrangement for simple hydrophobic surfaces like a flat graphene sheet, arranged mainly within a three-fold HB coordination with other water molecules (lacking a fourth neighbor in the direction of the hydrophobic surface) with HBs internal geometries improved with respect to typical bulk values [15].

The conclusion that transpires from Fig. 1 is at first glance paradoxical since it states that water distances itself more from the water-exposed bond than from the well-wrapped bond. The paradox may be resolved by examining the sub-nanoscale topographic patterns (curvature radius θ<10Å) on the protein surface, focusing on the θ-spectrum of cavities shaped by the residues paired by the BHBs. To determine interfacial θ-values, the solvent-accessible envelope of the protein surface [16], [17] is covered by a minimal set of water-confining osculating (first-order contact) spheres whose radius θ coincides with the envelope curvature radius at the point of contact. Thus, at a first-order contact point, all directional first derivatives of both osculating sphere and surface envelope coincide. Concave regions on the protein surface are characterized by θ>0, while θ<0 defines convex regions.

**Figure 2:**
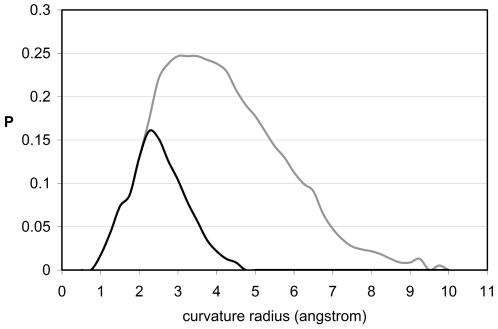
Surface ruggedness and backbone exposure in soluble proteins. Probability distribution of sub-nanoscale curvature radii of regions on the protein surface in the vicinity of BHBs (grey line), with the EBHB contribution represented by the black line. A region on the protein surface is defined as being in the vicinity of a BHB if it is shaped by solvent-exposed groups contained within a sphere of radius 3Å centered at the baricenter of the BHB. The curvature radius of a point on the protein water-exposed enveloping surface [16], [17] is defined as the radius of a first-order contact (osculating) sphere.

**Figure 3:**
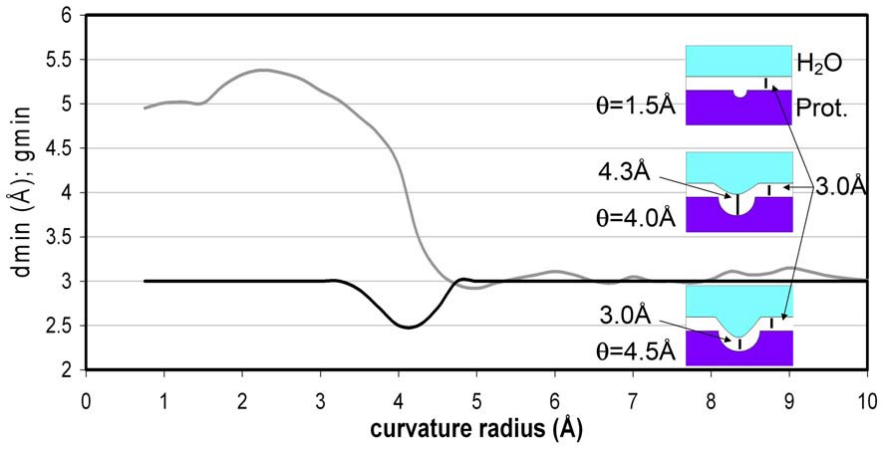
Surface curvature dependence of minimum distance and coordination index for water molecule closest to BHB. Distance to BHB of closest water molecule (d_min_ in angstroms, grey) and HB-coordination of the molecule (dimensionless g_min_, black) plotted as a function of the curvature radius (θ) of the protein surface vicinal to the BHB. The parameters d_min_, g_min_ are computed as averages over all BHBs with the same θ-value. The latter are coarse grained to ¼ of an angstrom. The inset describes the extent of water penetration for three illustrative θ-values.

Remarkably, as shown in Fig. 2, the range θ<2.25Å of the overall θ-distribution is completely dominated by the contribution from residues paired by EBHBs and completely absent from the θ-spectrum of well-wrapped BHBs. The distribution of θ-values for EBHBs peaks at ∼θ = 2.25Å and presents a negligibly small population for θ>4Å. Thus, the exposure of backbone hydrogen bonds in native folds occurs invariably through the formation of small cavities that, in turn, form exclusively around EBHBs. These sub-nanometer voids do not favor water penetration (Fig. 3), thus providing a geometrical hindrance to an otherwise accessible BHB. Such water exclusion can be rationalized since a water molecule can be accommodated at ∼3Å distance from an EBHB inside its typical size cavities, but this could only occur at the cost of curtailing hydrogen bonding with other water molecules. As shown in Fig. 3, water only penetrates the cavity if it can do so together with its coordination domain of hydrogen-bonded neighbors, so that its water-water coordination number (g, rigorously defined in Methods) is maintained and hardly ever lies below g = 3. The penetration of the well-coordinated interfacial water (g≅3) only occurs in the range θ≥3.5Å whereas the closest water molecule reaches its hydration-enabling distance d_min_ = 3Å to the BHB for curvature range θ≥4.5Å. Thus, hydration of an EBHB is practically forbidden since the fraction of EBHBs with θ≥4.5Å is less than 1% (Fig. 2). On the other hand, larger penetration-enabling cavity sizes in the range θ≥4.5Å are well represented in the θ-spectrum of well-wrapped BHBs (Fig. 2). However, in this case, water attack on the intramolecular interaction is intrinsically forbidden by the complete desolvation shell around the bond, made up of nearby side-chain nonpolar groups [4], [6], [18].

The topography-related dryness of EBHBs described in Figs. 1, 2 and 3 appears to be a geometric signature of native folds. The most extreme illustration of this kind of seal is provided by a potassium channel neurotoxin with PDB entry 1QUZ [9], where all 17 BHBs are actually exposed, while the θ-spectrum lies within the penetration-forbidding range θ = 2.36±1.16Å (Table S1). In fact, this protein has the lowest average curvature radius of all PDB-reported proteins.

**Figure 4:**
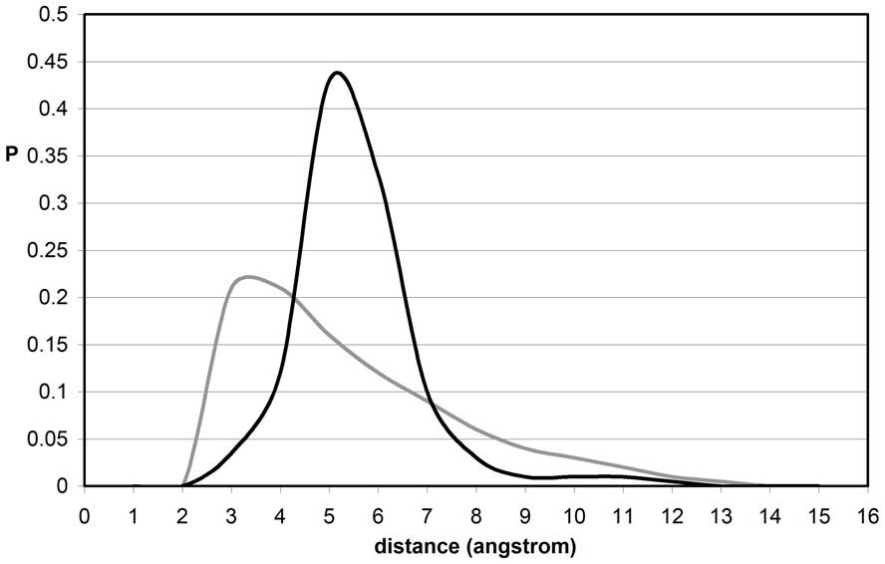
Distances of exposed BHBs to interfacial water for native folds and misfolded proteins. Probability distribution of distances from EBHBs of native folds (black line) and from EBHBs of misfolded proteins (grey line) to the closest surrounding water molecule. The datapoints on the grey line were obtained for 25 equilibrated misfolded structures [10], [19]. The distributions were obtained from the equilibrium values of the parameter d_min_ for 31,072 BHBs and 17,108 EBHBs from a dataset of misfolded structures obtained by threading a PDB-reported protein onto the structure of another [10].

**Figure 5:**
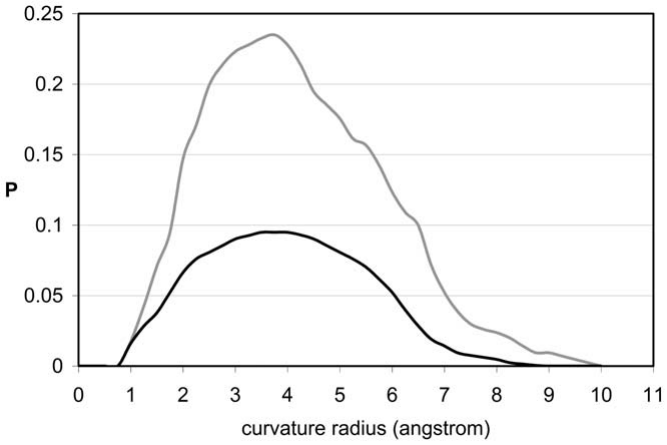
Surface ruggedness and backbone exposure for misfolded proteins. Probability distribution of sub-nanoscale curvature radii of regions on the surface of misfolded proteins in the vicinity of BHBs (grey line), with the EBHB contribution represented by the black line.

To properly assess the native-like nature of the topographic seal, we examined a database of misfolded proteins constructed using the Holm-Sander threading of PDB-reported proteins onto a different structure [19] followed by equilibration within the surrounding water (Methods) [10]. In contrast with native folds, the EBHBs in misfolded structures present closer water molecules (most probable distance d_min_∼3Å) and hence are unlikely to prevail due to competing backbone hydration (Fig. 4). This picture is reinforced by the far broader θ-spectrum for EBHBs in misfolded proteins when compared with the native folds (compare the black curves in Figs. 2 and 5). Thus, about 40% of EBHBs in misfolded proteins lie in the range θ≥4.5Å and hence are susceptible of being attacked by water due to the enabled water penetration (Fig. 3). Overall, we may say that the relative lack of sub-nanoscale ruggedness of misfolded structures when compared with native folds makes the former far more vulnerable to water attack.

The results from Fig. 3 underscore a spatially dependent least-action principle operative for water that is not hydrogen bonded to the protein. Thus, spatially related differences in hydrogen-bonding coordination are penalized as increments in the action. In rigorous terms, let A[g] denote the action functional, with g: *R* ^3^→*R* indicating the water-coordination scalar field (rigorously defined in Methods), so that vector position **r** for a water molecule is associated with coordination number g(**r**). Then, the action A[g] becomes
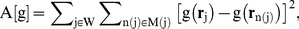
(1)where j, **r**_j_ indicate an individual water molecule and its respective position, W is the set of water molecules, n(j) denotes a neighboring water molecule coordinated to the *j*th molecule, and M(j) is the collection of neighboring molecules around the *j*th molecule. Thus, A[g] is minimized by a discrete scalar field g = {g_j_}_j∈W_ that reflects the tendency of interfacial water to retain its g = 3 coordination (cf. Fig. 3), and the general low resilience of water to lose more than a single hydrogen bond.

According to eq. 1, any solvent configuration that introduces a change in coordination number between neighboring molecules increases the action A[g] and hence becomes energetically penalized. Thus, the action dictates the near-constancy of g_min_ across the entire curvature spectrum (Fig. 3). To achieve this constancy, backbone solvation becomes only possible if the curvature of the cavity enables the solvating molecule to penetrate while retaining the coordination level of the interface (g = 3). Otherwise, the water molecules do not penetrate the cavity as they need to maintain their interfacial coordination level g = 3.

This work explores the real possibility of water-tight protein structures that nevertheless expose the backbone, a paradoxical situation since structural integrity of soluble proteins requires the sealing of backbone hydrogen bonds. For proteins with exposed backbone, the seal is shown to be maintained through a tightly fine-tuned distribution of sub-nanoscale curvatures of the protein enveloping surface that befits the low resilience of interfacial water in regards to retaining its coordination number (g = 3). Thus, the cavities around exposed backbone hydrogen bonds are too small to enable penetration of a g = 3 water molecule. The structural seal thus works by adapting the sub-nanoscale ruggedness of the protein surface to a spatially dependent least-action of water. This geometric adaptation tells apart native from misfolded structures, and hence introduces a physical constraint guiding the design of loose peptide-based materials that must remain water-tight to maintain structural integrity. Thus, the design concept introduced in this work will likely stimulate further endeavors in biomolecular engineering.

## Methods

### Identifying exposed backbone hydrogen bonds

A backbone hydrogen bond (BHB) is geometricaly defined [4], [6], [7], [18] as an interaction between a backbone amide and carbonyl, with N-O distance <3.2Å and angle a_HB_ between N-H and O = C bonds satisfying 120°≤a_HB_≤180°. A special kind of structural deficiency, the exposed backbone hydrogen bond (EBHB) is identified in soluble proteins with PDB-reported structure [4], [6], [18]. Thus, the extent of hydrogen bond protection is determined directly from atomic coordinates. This parameter, denoted ρ, is given by the number of side-chain carbonaceous nonpolar groups (CH_n_, n = 0, 1, 2, 3) contained within a desolvation domain that represents the hydrogen-bond local environment. This domain is defined as the reunion of two intersecting spheres of fixed radius (∼thickness of three water layers) centered at the α-carbons of the residues paired by the hydrogen bond. In structures of PDB-reported soluble proteins, backbone hydrogen bonds are protected on average by ρ = 26.6±7.5 side-chain nonpolar groups for a desolvation sphere of radius 6Å. Thus, structural deficiencies lie in the tail of the protection distribution, i.e. their microenvironment contains 19 or fewer nonpolar groups, so their ρ-value is below the mean ( = 26.6) minus one standard deviation ( = 7.5). While the statistics on ρ-values for backbone hydrogen bonds vary with the radius, the tails of the distribution remain invariant, thus enabling a robust identification of structural deficiencies [4], [18]. Of the 377,116 BHBs examined in a dataset of 2661 PDB-reported uncomplexed proteins (Table S1), 97,120 were found to be EBHBs, fulfilling ρ≤19.

### Equilibrium parameters of local hydration

The solvent structure was described by the scalar field g = g(**r**) that assigns to each position vector **r** a scalar value indicating the expected hydrogen-bond coordination of a water molecule situated within a sphere centered at position **r** with radius 2.5Å (the thickness of single water layer). The coordination indicates the number of hydrogen-bonding neighboring water molecules. The expected g(**r**) value was computed as a time average over solvent configurations determined by molecular dynamics over a 100ns-period after the initial PDB-reported structure was equilibrated with the solvent (see below). A water-water hydrogen bond was defined by the geometric constraints: O-O distance <3.2Å and O-H-O angle a_HB_ satisfying 120°≤a_HB_≤180°. Local hydration patterns were described by the equilibrium parameters d_min_, g_min_, representing respectively the minimum distance between the oxygens of backbone carbonyl and solvating water molecule, and the coordination of the water molecule that realizes d_min_. The equilibrium parameters d_min_, g_min_ were obtained from classical MD trajectories generated using the GROMACS 3.0 package [20]. The initial state of the trajectories consisted on the PDB structure embedded in a pre-equilibrated cell of explicitly represented water molecules and counterions [13], [14]. The PDB structures for an exhaustive nonredundant set of 2661 monomeric uncomplexed proteins lacking prosthetic groups or ion coordination (Table S1, maximum chain length N = 1,290) were used to generate the statistics reported. Each MD trajectory spanning 300ps was generated adopting an integration time step of 2fs in an NPT ensemble with box size 20^3^ nm^3^ and periodic boundary conditions [20]. The box size was calibrated so that the solvation shell extended at least 7.5Å (∼thickness of three water layers) from the protein surface at all times. The box size was determined taking into account the unit-cell dimensions of even the largest monomeric protein, the *Botulinum* neurotoxin (PDB.1S0G, N = 1,290), with maximum cell dimension b = 123.12Å. The long-range electrostatics were treated using the Particle Mesh Ewald (PME) summation method [21]. A Nose-Hoover thermostat [22] was used to maintain the temperature at 300K, and a Tip3P water model with OPLS (Optimized Potential for Liquid Simulations) force field was adopted [13], [14]. A barostat scheme was maintained through a dedicated routine with the pressure held constant at 1 atm. using a weak-coupling algorithm [23]. After 300ns equilibration, the position of the closest water molecule relative to the carbonyl oxygen within a BHB was determined together with its g-value, yielding the hydration parameters d_min_, g_min_. Across the exhaustive database of monomeric free proteins with sustainable autonomous structure, the RMSD between the solvent-equilibrated structure and the starting PDB structure was found to be <1.0Å for backbone atoms and <1.5Å when side chains are included.

## Supporting Information

Table S1 Exhaustive nonredundant dataset of 2661 monomeric uncomplexed PDB-reported proteins lacking prosthetic groups or ion coordination.
(4.10 MB DOC)
