# Correction: Sub-Nanoscale Surface Ruggedness Provides a Water-Tight Seal for Exposed Regions in Soluble Protein Structure

**DOI:** 10.1371/annotation/c17f2565-d129-4bb6-bb94-790ca1c6bb61

**Published:** 2013-12-30

**Authors:** Erica Schulz, Marisa Frechero, Gustavo Appignanesi, Ariel Fernández

The authors would like to provide additional information in relation to the methodology employed for the analyses reported in the article. This additional information is outlined here: http://www.plosone.org/corrections/pone.0012844.001.cn.doc

