# Supplementary material for: Sub-Nanoscale Surface Ruggedness Provides a Water-Tight Seal for Exposed Regions in Soluble Protein Structure
Source: PLoS One. 2010 Sep 17;5(9):e12844. doi: 10.1371/journal.pone.0012844 (PMC2941462; doi:10.1371/journal.pone.0012844)
Supplement: Table S1 — Exhaustive nonredundant dataset of 2661 monomeric uncomplexed PDB-reported proteins lacking prosthetic groups or ion coordination. (4.10 MB DOC) [file pone.0012844.s001.doc]

**Supplementary Information**

**Sub-nanoscale surface ruggedness provides a water-tight seal for**

**exposed regions in soluble protein structure**

Erica P. Schulz^1#^, Marisa A. Frechero^1#^, , Gustavo A. Appignanesi^1^ and

Ariel Fernández^2,3*^

^#^ Equal contribution

^1^ Sección Fisicoquímica, INQUISUR-UNS-CONICET and Departamento de Química, Universidad Nacional del Sur, Avda. Alem 1253, 8000 Bahía Blanca, Argentina

^2^ Department of Bioengineering, Rice University, Houston, TX 77005

^3^Department of Computer Science, The University of Chicago, Chicago, IL 60637

* Corresponding author, Phone: 713 348 3681; FAX: 713 348 3699;

Email: [arifer@rice.edu](mailto:arifer@rice.edu)

**Table S1.** Exhaustive nonredundant dataset of 2661 monomeric uncomplexed PDB-reported proteins lacking prosthetic groups or ion coordination. The dataset yields 377,116 BHBs and 97,120 EBHBs. Each protein is described by its PDB accession entry, number of BHBs, number of EBHBs and average sub-nanoscale curvature radius (<θ>).

| PDB entry | chain length | BHBs | EBHBs | <θ> |
| --- | --- | --- | --- | --- |
| 1QUZ | 34 | 17 | 17 | 2.36 |
| 1K9B | 58 | 22 | 22 | 2.39 |
| 2P4L | 46 | 11 | 11 | 2.40 |
| 1DF6 | 30 | 13 | 12 | 2.64 |
| 1AG7 | 34 | 12 | 11 | 2.67 |
| 1CMR | 31 | 10 | 9 | 2.73 |
| 1DW5 | 25 | 9 | 8 | 2.77 |
| 1FYG | 25 | 9 | 8 | 2.77 |
| 1HVW | 25 | 9 | 8 | 2.77 |
| 2HM5 | 31 | 9 | 8 | 2.77 |
| 2B38 | 31 | 17 | 15 | 2.79 |
| 1YZ2 | 26 | 8 | 7 | 2.82 |
| 1OMN | 26 | 8 | 7 | 2.82 |
| 1D5Q | 27 | 24 | 21 | 2.82 |
| 1Y2P | 34 | 16 | 14 | 2.82 |
| 1OIG | 24 | 8 | 7 | 2.82 |
| 1I8Y | 28 | 8 | 7 | 2.82 |
| 1TTK | 25 | 15 | 13 | 2.85 |
| 1IB9 | 34 | 15 | 13 | 2.85 |
| 2DDL | 33 | 22 | 19 | 2.86 |
| 1ORX | 24 | 7 | 6 | 2.88 |
| 1MVZ | 62 | 14 | 12 | 2.88 |
| 1FWO | 35 | 7 | 6 | 2.88 |
| 1CHL | 36 | 20 | 17 | 2.91 |
| 1AGT | 38 | 20 | 17 | 2.91 |
| 1MVI | 25 | 13 | 11 | 2.92 |
| 1WPD | 34 | 13 | 11 | 2.92 |
| 1V4Q | 26 | 13 | 11 | 2.92 |
| 1OMC | 27 | 13 | 11 | 2.92 |
| 1KCP | 27 | 13 | 11 | 2.92 |
| 2JTU | 38 | 13 | 11 | 2.92 |
| 1NBJ | 30 | 19 | 16 | 2.94 |
| 2D56 | 53 | 31 | 26 | 2.95 |
| 1ZUV | 30 | 12 | 10 | 2.97 |
| 2AJW | 22 | 18 | 15 | 2.97 |
| 2JRY | 46 | 18 | 15 | 2.97 |
| 1L4V | 40 | 29 | 24 | 2.99 |
| 1TXM | 34 | 17 | 14 | 3.01 |
| 1JLO | 24 | 11 | 9 | 3.02 |
| 1V91 | 37 | 11 | 9 | 3.02 |
| 1G26 | 31 | 11 | 9 | 3.02 |
| 2AK0 | 23 | 16 | 13 | 3.05 |
| 1G9P | 45 | 16 | 13 | 3.05 |
| 1HEV | 43 | 21 | 17 | 3.06 |
| 1H34 | 83 | 21 | 17 | 3.06 |
| 1SCY | 31 | 26 | 21 | 3.06 |
| 2EEM | 34 | 31 | 25 | 3.07 |
| 1ZPX | 21 | 5 | 4 | 3.09 |
| 1SIS | 35 | 24 | 19 | 3.12 |
| 1Q7J | 68 | 19 | 15 | 3.13 |
| 1NBL | 46 | 33 | 26 | 3.13 |
| 1TT3 | 25 | 14 | 11 | 3.14 |
| 1KQH | 38 | 14 | 11 | 3.14 |
| 1ZNU | 29 | 14 | 11 | 3.14 |
| 1ZWU | 30 | 14 | 11 | 3.14 |
| 2IT8 | 30 | 9 | 7 | 3.17 |
| 1M2S | 37 | 27 | 21 | 3.17 |
| 2JYE | 72 | 22 | 17 | 3.19 |
| 1ICA | 40 | 22 | 17 | 3.19 |
| 6HIR | 65 | 13 | 10 | 3.20 |
| 1ERP | 38 | 30 | 23 | 3.21 |
| 1RJI | 31 | 17 | 13 | 3.22 |
| 2K1I | 32 | 17 | 13 | 3.22 |
| 2K4U | 37 | 17 | 13 | 3.22 |
| 2KTX | 38 | 17 | 13 | 3.22 |
| 1QKY | 38 | 21 | 16 | 3.23 |
| 1C56 | 40 | 21 | 16 | 3.23 |
| 1TSK | 35 | 25 | 19 | 3.24 |
| 2NY8 | 40 | 25 | 19 | 3.24 |
| 1B45 | 14 | 4 | 3 | 3.27 |
| 1Q2J | 22 | 4 | 3 | 3.27 |
| 1DG2 | 15 | 4 | 3 | 3.27 |
| 1KG1 | 60 | 23 | 17 | 3.31 |
| 1KMX | 55 | 23 | 17 | 3.31 |
| 2UVS | 38 | 19 | 14 | 3.32 |
| 4HIR | 65 | 19 | 14 | 3.32 |
| 1T0W | 32 | 15 | 11 | 3.33 |
| 2G9L | 37 | 37 | 27 | 3.35 |
| 1N8M | 38 | 22 | 16 | 3.36 |
| 1BH4 | 30 | 11 | 8 | 3.36 |
| 1MMC | 30 | 11 | 8 | 3.36 |
| 1VFI | 95 | 73 | 53 | 3.36 |
| 2ERI | 31 | 18 | 13 | 3.37 |
| 1AZK | 36 | 18 | 13 | 3.37 |
| 1PX9 | 42 | 25 | 18 | 3.38 |
| 1MII | 16 | 7 | 5 | 3.40 |
| 1TR6 | 27 | 14 | 10 | 3.40 |
| 1HA8 | 51 | 42 | 30 | 3.40 |
| 2B68 | 43 | 21 | 15 | 3.40 |
| 1LMR | 35 | 7 | 5 | 3.40 |
| 1SCO | 38 | 28 | 20 | 3.40 |
| 1UDK | 51 | 14 | 10 | 3.40 |
| 1ADX | 40 | 7 | 5 | 3.40 |
| 1WQD | 27 | 21 | 15 | 3.40 |
| 1KVF | 16 | 7 | 5 | 3.40 |
| 1UEO | 63 | 21 | 15 | 3.40 |
| 1AZ6 | 36 | 17 | 12 | 3.43 |
| 1L1I | 84 | 27 | 19 | 3.44 |
| 1NE5 | 42 | 27 | 19 | 3.44 |
| 2IKE | 54 | 37 | 26 | 3.44 |
| 2H8S | 16 | 10 | 7 | 3.45 |
| 1F3K | 26 | 10 | 7 | 3.45 |
| 2HGO | 27 | 10 | 7 | 3.45 |
| 1Q9B | 43 | 20 | 14 | 3.45 |
| 1WKX | 43 | 20 | 14 | 3.45 |
| 1Q7I | 68 | 20 | 14 | 3.45 |
| 1BKT | 37 | 20 | 14 | 3.45 |
| 1JU8 | 37 | 10 | 7 | 3.45 |
| 1XSW | 38 | 10 | 7 | 3.45 |
| 2YS0 | 56 | 20 | 14 | 3.45 |
| 1GK5 | 49 | 10 | 7 | 3.45 |
| 1L3Y | 41 | 13 | 9 | 3.48 |
| 1E4R | 35 | 13 | 9 | 3.48 |
| 2NY9 | 41 | 26 | 18 | 3.48 |
| 1R02 | 33 | 26 | 18 | 3.48 |
| 1HYK | 46 | 16 | 11 | 3.50 |
| 1WT8 | 31 | 16 | 11 | 3.50 |
| 2K2X | 75 | 25 | 17 | 3.53 |
| 2A2B | 41 | 25 | 17 | 3.53 |
| 2PJG | 68 | 28 | 19 | 3.53 |
| 1MXN | 15 | 9 | 6 | 3.58 |
| 1SOP | 24 | 9 | 6 | 3.58 |
| 1V56 | 34 | 18 | 12 | 3.58 |
| 1WO1 | 17 | 6 | 4 | 3.58 |
| 2GLG | 32 | 47 | 31 | 3.60 |
| 2FD9 | 46 | 35 | 23 | 3.61 |
| 2GLH | 32 | 35 | 23 | 3.61 |
| 2E3E | 45 | 26 | 17 | 3.62 |
| 1LXE | 101 | 49 | 32 | 3.63 |
| 1NEA | 61 | 23 | 15 | 3.63 |
| 1WQC | 26 | 20 | 13 | 3.64 |
| 1G6M | 62 | 20 | 13 | 3.64 |
| 1FK4 | 93 | 94 | 61 | 3.64 |
| 1FVL | 70 | 17 | 11 | 3.65 |
| 1UT3 | 38 | 17 | 11 | 3.65 |
| 2HLG | 39 | 17 | 11 | 3.65 |
| 1UVB | 91 | 87 | 56 | 3.66 |
| 1MYN | 44 | 28 | 18 | 3.66 |
| 1ATB | 62 | 28 | 18 | 3.66 |
| 1UOY | 64 | 39 | 25 | 3.67 |
| 1LGL | 36 | 25 | 16 | 3.67 |
| 1FK6 | 93 | 86 | 55 | 3.67 |
| 1ULN | 84 | 36 | 23 | 3.68 |
| 1FK2 | 93 | 91 | 58 | 3.68 |
| 2GX1 | 29 | 11 | 7 | 3.69 |
| 1RMK | 31 | 11 | 7 | 3.69 |
| 2K10 | 32 | 30 | 19 | 3.70 |
| 1ZA8 | 31 | 19 | 12 | 3.70 |
| 1N4Y | 68 | 19 | 12 | 3.70 |
| 1FQQ | 41 | 19 | 12 | 3.70 |
| 1CXR | 46 | 19 | 12 | 3.70 |
| 1FFM | 46 | 19 | 12 | 3.70 |
| 1XV3 | 47 | 19 | 12 | 3.70 |
| 1ATE | 62 | 27 | 17 | 3.71 |
| 2CK4 | 38 | 27 | 17 | 3.71 |
| 1SH1 | 48 | 27 | 17 | 3.71 |
| 1FK3 | 93 | 91 | 57 | 3.72 |
| 1MZM | 93 | 91 | 57 | 3.72 |
| 1FK1 | 93 | 91 | 57 | 3.72 |
| 1WO0 | 17 | 8 | 5 | 3.73 |
| 1FJN | 39 | 24 | 15 | 3.73 |
| 2GWP | 32 | 16 | 10 | 3.73 |
| 1ZJQ | 34 | 8 | 5 | 3.73 |
| 1FK5 | 93 | 93 | 58 | 3.73 |
| 1FK7 | 93 | 90 | 56 | 3.74 |
| 1OZZ | 44 | 29 | 18 | 3.74 |
| 1CW6 | 37 | 29 | 18 | 3.74 |
| 1OHN | 43 | 29 | 18 | 3.74 |
| 1B8W | 42 | 21 | 13 | 3.75 |
| 1F7E | 46 | 21 | 13 | 3.75 |
| 1MZL | 93 | 89 | 55 | 3.75 |
| 2A05 | 57 | 34 | 21 | 3.75 |
| 2JTO | 75 | 34 | 21 | 3.75 |
| 1OG7 | 43 | 34 | 21 | 3.75 |
| 1AV3 | 27 | 13 | 8 | 3.76 |
| 1IE6 | 33 | 13 | 8 | 3.76 |
| 1RYV | 35 | 13 | 8 | 3.76 |
| 1ATD | 62 | 26 | 16 | 3.76 |
| 2Z3S | 39 | 31 | 19 | 3.77 |
| 1JMP | 46 | 31 | 19 | 3.77 |
| 1GPT | 47 | 36 | 22 | 3.78 |
| 1ORL | 46 | 41 | 25 | 3.78 |
| 2NLS | 36 | 23 | 14 | 3.79 |
| 1HP2 | 37 | 23 | 14 | 3.79 |
| 2SH1 | 48 | 28 | 17 | 3.79 |
| 1ERY | 39 | 38 | 23 | 3.80 |
| 2IKD | 66 | 38 | 23 | 3.80 |
| 1EHD | 89 | 48 | 29 | 3.80 |
| 1N0C | 10 | 5 | 3 | 3.82 |
| 1UVA | 91 | 72 | 43 | 3.83 |
| 1EN2 | 89 | 47 | 28 | 3.83 |
| 1ENM | 89 | 47 | 28 | 3.83 |
| 1PE4 | 67 | 32 | 19 | 3.84 |
| 2VGH | 55 | 27 | 16 | 3.85 |
| 1APQ | 53 | 27 | 16 | 3.85 |
| 1PJV | 32 | 22 | 13 | 3.85 |
| 1TLE | 58 | 17 | 10 | 3.86 |
| 2FD7 | 46 | 34 | 20 | 3.86 |
| 2H9X | 47 | 17 | 10 | 3.86 |
| 2CQ7 | 49 | 17 | 10 | 3.86 |
| 4TGF | 50 | 17 | 10 | 3.86 |
| 1P00 | 44 | 29 | 17 | 3.87 |
| 1BKU | 32 | 29 | 17 | 3.87 |
| 1EMX | 30 | 12 | 7 | 3.88 |
| 1J5J | 36 | 24 | 14 | 3.88 |
| 1L5C | 92 | 43 | 25 | 3.89 |
| 1EPH | 53 | 19 | 11 | 3.89 |
| 1AZJ | 36 | 19 | 11 | 3.89 |
| 1HRE | 67 | 19 | 11 | 3.89 |
| 1TOZ | 116 | 45 | 26 | 3.90 |
| 1MM0 | 36 | 26 | 15 | 3.90 |
| 1ATA | 62 | 26 | 15 | 3.90 |
| 1KMA | 55 | 26 | 15 | 3.90 |
| 1C2A | 120 | 33 | 19 | 3.91 |
| 1EWW | 90 | 33 | 19 | 3.91 |
| 2AAP | 34 | 14 | 8 | 3.92 |
| 1NIY | 35 | 14 | 8 | 3.92 |
| 1D1H | 35 | 14 | 8 | 3.92 |
| 1BGK | 37 | 28 | 16 | 3.92 |
| 1ZUE | 42 | 14 | 8 | 3.92 |
| 1NH5 | 60 | 35 | 20 | 3.92 |
| 1EFE | 60 | 44 | 25 | 3.93 |
| 2FMC | 82 | 30 | 17 | 3.94 |
| 2FQC | 25 | 23 | 13 | 3.94 |
| 1L5D | 92 | 39 | 22 | 3.95 |
| 1AXH | 37 | 16 | 9 | 3.95 |
| 1CHV | 60 | 16 | 9 | 3.95 |
| 2JMV | 95 | 32 | 18 | 3.95 |
| 1CW5 | 48 | 32 | 18 | 3.95 |
| 1VNB | 65 | 25 | 14 | 3.96 |
| 1OHM | 44 | 25 | 14 | 3.96 |
| 2PM1 | 30 | 9 | 5 | 3.98 |
| 1QK6 | 33 | 9 | 5 | 3.98 |
| 1IVA | 48 | 9 | 5 | 3.98 |
| 1P0A | 44 | 36 | 20 | 3.98 |
| 1ZAQ | 44 | 9 | 5 | 3.98 |
| 2K6A | 68 | 38 | 21 | 3.99 |
| 1HPW | 129 | 67 | 37 | 3.99 |
| 1HRF | 67 | 20 | 11 | 4.00 |
| 1TER | 21 | 11 | 6 | 4.02 |
| 1QK7 | 32 | 11 | 6 | 4.02 |
| 1B9W | 95 | 44 | 24 | 4.02 |
| 1HAE | 63 | 33 | 18 | 4.02 |
| 1HAF | 63 | 33 | 18 | 4.02 |
| 1SS3 | 50 | 33 | 18 | 4.02 |
| 3EZM | 101 | 57 | 31 | 4.02 |
| 1BMR | 67 | 35 | 19 | 4.03 |
| 1SIY | 91 | 70 | 38 | 4.03 |
| 2E3G | 40 | 24 | 13 | 4.03 |
| 1IP0 | 50 | 24 | 13 | 4.03 |
| 1SS2 | 68 | 24 | 13 | 4.03 |
| 1JXW | 46 | 37 | 20 | 4.03 |
| 1JXY | 46 | 37 | 20 | 4.03 |
| 1CRN | 46 | 37 | 20 | 4.03 |
| 1JXT | 46 | 37 | 20 | 4.03 |
| 1CNR | 46 | 37 | 20 | 4.03 |
| 1XHH | 91 | 37 | 20 | 4.03 |
| 1MR0 | 34 | 13 | 7 | 4.04 |
| 1KQI | 38 | 13 | 7 | 4.04 |
| 1RYG | 35 | 13 | 7 | 4.04 |
| 2NSV | 52 | 26 | 14 | 4.04 |
| 1WXN | 42 | 28 | 15 | 4.05 |
| 1N4N | 47 | 30 | 16 | 4.06 |
| 1S8K | 30 | 15 | 8 | 4.06 |
| 1EWS | 32 | 15 | 8 | 4.06 |
| 1VEX | 56 | 17 | 9 | 4.07 |
| 2YRA | 74 | 17 | 9 | 4.07 |
| 1FH3 | 67 | 36 | 19 | 4.08 |
| 2JXZ | 33 | 36 | 19 | 4.08 |
| 1AFH | 93 | 74 | 39 | 4.08 |
| 3BBG | 40 | 19 | 10 | 4.09 |
| 1VTX | 42 | 19 | 10 | 4.09 |
| 2GW9 | 32 | 19 | 10 | 4.09 |
| 1OAV | 48 | 19 | 10 | 4.09 |
| 2EYB | 46 | 38 | 20 | 4.09 |
| 1IMT | 80 | 38 | 20 | 4.09 |
| 1P9J | 54 | 19 | 10 | 4.09 |
| 1SZL | 61 | 19 | 10 | 4.09 |
| 1XI7 | 61 | 19 | 10 | 4.09 |
| 2EC3 | 68 | 19 | 10 | 4.09 |
| 2QT4 | 95 | 40 | 21 | 4.09 |
| 2JQ8 | 53 | 21 | 11 | 4.10 |
| 1JXC | 68 | 21 | 11 | 4.10 |
| 1EPG | 53 | 21 | 11 | 4.10 |
| 3BT4 | 86 | 44 | 23 | 4.10 |
| 1JGK | 66 | 23 | 12 | 4.10 |
| 2E2F | 41 | 23 | 12 | 4.10 |
| 1BV2 | 91 | 69 | 36 | 4.10 |
| 1HFI | 62 | 23 | 12 | 4.10 |
| 2JZM | 53 | 25 | 13 | 4.11 |
| 1Z1X | 64 | 25 | 13 | 4.11 |
| 1WHF | 86 | 25 | 13 | 4.11 |
| 1L6H | 69 | 52 | 27 | 4.11 |
| 1UZQ | 162 | 79 | 41 | 4.11 |
| 1YVA | 46 | 27 | 14 | 4.11 |
| 1DZ7 | 92 | 27 | 14 | 4.11 |
| 2JQP | 65 | 29 | 15 | 4.12 |
| 1W6B | 73 | 29 | 15 | 4.12 |
| 1CC5 | 83 | 60 | 31 | 4.12 |
| 2SN3 | 65 | 31 | 16 | 4.12 |
| 1IY5 | 54 | 31 | 16 | 4.12 |
| 1HKY | 86 | 35 | 18 | 4.13 |
| 1VJW | 60 | 35 | 18 | 4.13 |
| 1J5I | 122 | 72 | 37 | 4.13 |
| 1CBN | 46 | 37 | 19 | 4.13 |
| 1JXX | 46 | 37 | 19 | 4.13 |
| 2E0H | 64 | 39 | 20 | 4.14 |
| 1T12 | 91 | 88 | 45 | 4.14 |
| 1NT3 | 119 | 47 | 24 | 4.14 |
| 1NOA | 113 | 61 | 31 | 4.15 |
| 2B5K | 19 | 4 | 2 | 4.18 |
| 1LUP | 31 | 6 | 3 | 4.18 |
| 1KCO | 21 | 8 | 4 | 4.18 |
| 1WQB | 32 | 8 | 4 | 4.18 |
| 1ROO | 35 | 20 | 10 | 4.18 |
| 1HX2 | 60 | 24 | 12 | 4.18 |
| 2I1T | 36 | 12 | 6 | 4.18 |
| 1MPV | 12 | 6 | 3 | 4.18 |
| 1HD6 | 37 | 38 | 19 | 4.18 |
| 1KTX | 37 | 18 | 9 | 4.18 |
| 1CZ6 | 25 | 8 | 4 | 4.18 |
| 1BNB | 38 | 14 | 7 | 4.18 |
| 1AYJ | 51 | 32 | 16 | 4.18 |
| 2H1Z | 39 | 14 | 7 | 4.18 |
| 1DEC | 39 | 12 | 6 | 4.18 |
| 1MTX | 39 | 14 | 7 | 4.18 |
| 1ERC | 40 | 36 | 18 | 4.18 |
| 2DCW | 42 | 16 | 8 | 4.18 |
| 1CTX | 71 | 24 | 12 | 4.18 |
| 1TXB | 73 | 18 | 9 | 4.18 |
| 1U5M | 73 | 24 | 12 | 4.18 |
| 1I2U | 44 | 24 | 12 | 4.18 |
| 1LR9 | 74 | 38 | 19 | 4.18 |
| 1CCQ | 60 | 26 | 13 | 4.18 |
| 1ZAD | 60 | 24 | 12 | 4.18 |
| 1CRE | 60 | 20 | 10 | 4.18 |
| 1GJE | 15 | 10 | 5 | 4.18 |
| 1JE9 | 61 | 26 | 13 | 4.18 |
| 1QM7 | 61 | 26 | 13 | 4.18 |
| 2EYD | 46 | 32 | 16 | 4.18 |
| 2B3C | 64 | 30 | 15 | 4.18 |
| 1LQQ | 64 | 38 | 19 | 4.18 |
| 1LDL | 48 | 16 | 8 | 4.18 |
| 1ROF | 60 | 26 | 13 | 4.18 |
| 1BEG | 98 | 87 | 43 | 4.20 |
| 1CZ2 | 90 | 71 | 35 | 4.21 |
| 1SF6 | 129 | 61 | 30 | 4.21 |
| 1EI0 | 38 | 49 | 24 | 4.22 |
| 2EYA | 46 | 35 | 17 | 4.23 |
| 1EJG | 46 | 35 | 17 | 4.23 |
| 1SNB | 64 | 35 | 17 | 4.23 |
| 1QGB | 93 | 35 | 17 | 4.23 |
| 1EHS | 48 | 31 | 15 | 4.24 |
| 1JA4 | 129 | 62 | 30 | 4.24 |
| 1T50 | 58 | 29 | 14 | 4.24 |
| 2K3G | 102 | 29 | 14 | 4.24 |
| 2JXD | 62 | 29 | 14 | 4.24 |
| 1SRZ | 68 | 29 | 14 | 4.24 |
| 2CTX | 71 | 27 | 13 | 4.25 |
| 1YV8 | 46 | 27 | 13 | 4.25 |
| 2JVU | 104 | 54 | 26 | 4.25 |
| 1NOR | 61 | 25 | 12 | 4.25 |
| 2ERA | 62 | 25 | 12 | 4.25 |
| 1HZK | 110 | 50 | 24 | 4.25 |
| 1SF4 | 129 | 73 | 35 | 4.26 |
| 1DQB | 83 | 23 | 11 | 4.26 |
| 1I2V | 44 | 23 | 11 | 4.26 |
| 1NTX | 60 | 23 | 11 | 4.26 |
| 1FRA | 62 | 23 | 11 | 4.26 |
| 1APF | 49 | 23 | 11 | 4.26 |
| 2J4M | 100 | 46 | 22 | 4.26 |
| 1JTB | 91 | 69 | 33 | 4.26 |
| 1CBH | 36 | 21 | 10 | 4.27 |
| 1O5P | 113 | 61 | 29 | 4.27 |
| 2BBG | 40 | 19 | 9 | 4.28 |
| 2CBH | 36 | 19 | 9 | 4.28 |
| 1BIP | 122 | 76 | 36 | 4.28 |
| 3GF1 | 70 | 53 | 25 | 4.28 |
| 1QDP | 42 | 17 | 8 | 4.29 |
| 1NTN | 72 | 34 | 16 | 4.29 |
| 2K35 | 60 | 34 | 16 | 4.29 |
| 1CCM | 46 | 34 | 16 | 4.29 |
| 1JXU | 46 | 34 | 16 | 4.29 |
| 1EGF | 53 | 17 | 8 | 4.29 |
| 1TPG | 91 | 34 | 16 | 4.29 |
| 2ERW | 56 | 34 | 16 | 4.29 |
| 1Z2G | 69 | 51 | 24 | 4.29 |
| 1VIB | 55 | 49 | 23 | 4.29 |
| 2J4N | 100 | 49 | 23 | 4.29 |
| 1DS3 | 51 | 32 | 15 | 4.30 |
| 1SSZ | 34 | 32 | 15 | 4.30 |
| 1TAP | 60 | 32 | 15 | 4.30 |
| 1FXD | 58 | 32 | 15 | 4.30 |
| 1JA2 | 129 | 62 | 29 | 4.30 |
| 1TXA | 73 | 15 | 7 | 4.30 |
| 1Y1C | 48 | 30 | 14 | 4.30 |
| 1JZB | 66 | 30 | 14 | 4.30 |
| 1Z27 | 186 | 88 | 41 | 4.31 |
| 1PCP | 106 | 41 | 19 | 4.31 |
| 1DKC | 38 | 13 | 6 | 4.32 |
| 1IW4 | 55 | 26 | 12 | 4.32 |
| 1KFH | 74 | 39 | 18 | 4.32 |
| 1LR8 | 74 | 39 | 18 | 4.32 |
| 3CA7 | 52 | 26 | 12 | 4.32 |
| 1AZH | 36 | 13 | 6 | 4.32 |
| 2CKU | 90 | 39 | 18 | 4.32 |
| 1BZB | 32 | 26 | 12 | 4.32 |
| 1JA6 | 129 | 65 | 30 | 4.32 |
| 2RN9 | 67 | 39 | 18 | 4.32 |
| 2G0L | 122 | 52 | 24 | 4.32 |
| 1KX7 | 81 | 52 | 24 | 4.32 |
| 1SF7 | 129 | 63 | 29 | 4.33 |
| 2FYJ | 82 | 24 | 11 | 4.33 |
| 1DRS | 59 | 24 | 11 | 4.33 |
| 2CDX | 60 | 24 | 11 | 4.33 |
| 1MR6 | 68 | 22 | 10 | 4.35 |
| 1RMR | 64 | 22 | 10 | 4.35 |
| 1ACX | 108 | 44 | 20 | 4.35 |
| 1HYP | 80 | 64 | 29 | 4.35 |
| 1MID | 91 | 95 | 43 | 4.35 |
| 1F94 | 63 | 31 | 14 | 4.36 |
| 1QWV | 142 | 113 | 51 | 4.36 |
| 1W2Q | 127 | 82 | 37 | 4.36 |
| 2CG7 | 90 | 51 | 23 | 4.36 |
| 1IIY | 101 | 60 | 27 | 4.36 |
| 1IGL | 67 | 38 | 17 | 4.37 |
| 2GYZ | 105 | 47 | 21 | 4.38 |
| 1FYB | 111 | 56 | 25 | 4.38 |
| 1SFG | 129 | 65 | 29 | 4.38 |
| 2JTB | 33 | 9 | 4 | 4.38 |
| 1TFS | 60 | 27 | 12 | 4.38 |
| 1N2Y | 15 | 9 | 4 | 4.38 |
| 2GKT | 51 | 36 | 16 | 4.38 |
| 3EGF | 53 | 18 | 8 | 4.38 |
| 2IZ4 | 94 | 45 | 20 | 4.38 |
| 1PS2 | 60 | 18 | 8 | 4.38 |
| 2XBD | 87 | 36 | 16 | 4.38 |
| 1RYX | 686 | 327 | 145 | 4.39 |
| 1BW4 | 125 | 68 | 30 | 4.40 |
| 1VPI | 122 | 109 | 48 | 4.40 |
| 1Z6V | 49 | 25 | 11 | 4.40 |
| 1GH1 | 90 | 66 | 29 | 4.40 |
| 1LS8 | 142 | 123 | 54 | 4.40 |
| 1BXM | 99 | 98 | 43 | 4.40 |
| 1TI5 | 46 | 32 | 14 | 4.41 |
| 1BEI | 35 | 16 | 7 | 4.41 |
| 2EZN | 101 | 62 | 27 | 4.42 |
| 1WMT | 41 | 23 | 10 | 4.42 |
| 1TIH | 53 | 23 | 10 | 4.42 |
| 1FFJ | 60 | 23 | 10 | 4.42 |
| 2DWF | 34 | 23 | 10 | 4.42 |
| 1TUR | 56 | 23 | 10 | 4.42 |
| 2HLQ | 100 | 53 | 23 | 4.42 |
| 1H2P | 125 | 53 | 23 | 4.42 |
| 1GNC | 178 | 166 | 72 | 4.42 |
| 1CDS | 77 | 30 | 13 | 4.42 |
| 2HLR | 100 | 30 | 13 | 4.42 |
| 1BE2 | 91 | 67 | 29 | 4.43 |
| 1AHO | 64 | 37 | 16 | 4.43 |
| 1PTX | 64 | 37 | 16 | 4.43 |
| 3OVO | 56 | 37 | 16 | 4.43 |
| 1GKG | 136 | 44 | 19 | 4.43 |
| 1SV9 | 121 | 100 | 43 | 4.44 |
| 1SSL | 48 | 21 | 9 | 4.44 |
| 2B5B | 36 | 14 | 6 | 4.44 |
| 1H20 | 39 | 21 | 9 | 4.44 |
| 2ADX | 40 | 7 | 3 | 4.44 |
| 1CE3 | 54 | 21 | 9 | 4.44 |
| 1ERA | 62 | 21 | 9 | 4.44 |
| 1NRA | 63 | 35 | 15 | 4.44 |
| 1AHL | 49 | 14 | 6 | 4.44 |
| 1ZU3 | 66 | 35 | 15 | 4.44 |
| 2OTH | 121 | 96 | 41 | 4.45 |
| 2A6U | 129 | 75 | 32 | 4.45 |
| 2JVE | 91 | 47 | 20 | 4.45 |
| 1Q5F | 156 | 94 | 40 | 4.45 |
| 1H0Z | 68 | 40 | 17 | 4.45 |
| 1ZDD | 34 | 40 | 17 | 4.45 |
| 1MR4 | 47 | 33 | 14 | 4.46 |
| 1CN2 | 66 | 33 | 14 | 4.46 |
| 1L3H | 65 | 33 | 14 | 4.46 |
| 1CDR | 77 | 26 | 11 | 4.46 |
| 2JYY | 53 | 26 | 11 | 4.46 |
| 1OMT | 56 | 26 | 11 | 4.46 |
| 2K4R | 77 | 26 | 11 | 4.46 |
| 2O9X | 181 | 142 | 60 | 4.46 |
| 1E5G | 120 | 45 | 19 | 4.46 |
| 2P26 | 280 | 147 | 62 | 4.47 |
| 1UGL | 50 | 19 | 8 | 4.47 |
| 1BQT | 70 | 19 | 8 | 4.47 |
| 1Q8D | 108 | 93 | 39 | 4.48 |
| 2CBM | 112 | 62 | 26 | 4.48 |
| 2OLI | 121 | 105 | 44 | 4.48 |
| 2HDM | 92 | 43 | 18 | 4.48 |
| 1TV0 | 32 | 12 | 5 | 4.48 |
| 1LU8 | 34 | 12 | 5 | 4.48 |
| 2FUI | 62 | 24 | 10 | 4.48 |
| 1CRF | 60 | 24 | 10 | 4.48 |
| 1PCE | 60 | 36 | 15 | 4.48 |
| 1BET | 107 | 53 | 22 | 4.49 |
| 1RCL | 106 | 53 | 22 | 4.49 |
| 1HZL | 110 | 53 | 22 | 4.49 |
| 2IZ3 | 97 | 41 | 17 | 4.49 |
| 2JTK | 90 | 29 | 12 | 4.50 |
| 1KX2 | 81 | 58 | 24 | 4.50 |
| 1ZWT | 158 | 114 | 47 | 4.50 |
| 1T7A | 66 | 34 | 14 | 4.50 |
| 1T7B | 66 | 34 | 14 | 4.50 |
| 1E9T | 59 | 17 | 7 | 4.50 |
| 1LIP | 91 | 68 | 28 | 4.50 |
| 1NRB | 63 | 39 | 16 | 4.51 |
| 1H02 | 70 | 39 | 16 | 4.51 |
| 1BBG | 40 | 22 | 9 | 4.51 |
| 1WQK | 42 | 22 | 9 | 4.51 |
| 1QWQ | 124 | 88 | 36 | 4.51 |
| 1W1N | 33 | 22 | 9 | 4.51 |
| 2OUB | 121 | 98 | 40 | 4.52 |
| 2QVD | 121 | 103 | 42 | 4.52 |
| 1RL5 | 60 | 27 | 11 | 4.52 |
| 1FSC | 61 | 27 | 11 | 4.52 |
| 4OVO | 56 | 27 | 11 | 4.52 |
| 1LD6 | 58 | 27 | 11 | 4.52 |
| 1ZT3 | 80 | 54 | 22 | 4.52 |
| 1N02 | 102 | 59 | 24 | 4.52 |
| 1U3N | 162 | 32 | 13 | 4.52 |
| 2PVT | 121 | 101 | 41 | 4.52 |
| 1APJ | 74 | 42 | 17 | 4.53 |
| 2EDJ | 100 | 42 | 17 | 4.53 |
| 1WVZ | 104 | 47 | 19 | 4.53 |
| 2PMJ | 121 | 99 | 40 | 4.53 |
| 2QUE | 121 | 99 | 40 | 4.53 |
| 2COQ | 108 | 62 | 25 | 4.53 |
| 1Z2F | 121 | 82 | 33 | 4.54 |
| 1BEO | 98 | 107 | 43 | 4.54 |
| 1SRB | 21 | 10 | 4 | 4.55 |
| 2CRT | 60 | 25 | 10 | 4.55 |
| 1I02 | 60 | 25 | 10 | 4.55 |
| 1J5H | 122 | 65 | 26 | 4.55 |
| 2OTF | 121 | 103 | 41 | 4.55 |
| 1JA7 | 129 | 68 | 27 | 4.56 |
| 1SFB | 129 | 68 | 27 | 4.56 |
| 2PWS | 121 | 106 | 42 | 4.56 |
| 2OSH | 119 | 101 | 40 | 4.56 |
| 2PY0 | 120 | 91 | 36 | 4.56 |
| 1SN1 | 64 | 38 | 15 | 4.56 |
| 1GZZ | 70 | 38 | 15 | 4.56 |
| 1GZY | 70 | 38 | 15 | 4.56 |
| 1GZR | 70 | 38 | 15 | 4.56 |
| 1G91 | 77 | 38 | 15 | 4.56 |
| 1S62 | 106 | 71 | 28 | 4.57 |
| 1SVQ | 114 | 71 | 28 | 4.57 |
| 1PK2 | 90 | 33 | 13 | 4.57 |
| 1BRZ | 54 | 28 | 11 | 4.57 |
| 1TCP | 60 | 28 | 11 | 4.57 |
| 1X6R | 123 | 84 | 33 | 4.57 |
| 1X6P | 123 | 84 | 33 | 4.57 |
| 1COU | 85 | 51 | 20 | 4.57 |
| 2AFP | 129 | 51 | 20 | 4.57 |
| 2QHW | 121 | 105 | 41 | 4.58 |
| 1S6X | 34 | 18 | 7 | 4.59 |
| 2CCX | 60 | 18 | 7 | 4.59 |
| 1LQH | 65 | 36 | 14 | 4.59 |
| 1B9G | 57 | 36 | 14 | 4.59 |
| 1MKN | 59 | 18 | 7 | 4.59 |
| 1RMJ | 107 | 54 | 21 | 4.59 |
| 1X6Y | 123 | 85 | 33 | 4.59 |
| 1DZO | 123 | 85 | 33 | 4.59 |
| 1WWN | 69 | 49 | 19 | 4.59 |
| 2ZBH | 121 | 98 | 38 | 4.59 |
| 1PLO | 122 | 49 | 19 | 4.59 |
| 2HP8 | 68 | 80 | 31 | 4.59 |
| 1ERG | 70 | 31 | 12 | 4.59 |
| 2DDI | 70 | 31 | 12 | 4.59 |
| 1MH7 | 119 | 106 | 41 | 4.59 |
| 2NPL | 96 | 57 | 22 | 4.60 |
| 1TYK | 34 | 13 | 5 | 4.60 |
| 1KJ5 | 36 | 13 | 5 | 4.60 |
| 1CB9 | 60 | 26 | 10 | 4.60 |
| 1YTP | 53 | 26 | 10 | 4.60 |
| 1UUB | 56 | 26 | 10 | 4.60 |
| 1VYC | 65 | 26 | 10 | 4.60 |
| 1QLD | 50 | 26 | 10 | 4.60 |
| 1BO0 | 76 | 39 | 15 | 4.60 |
| 1PUB | 162 | 91 | 35 | 4.60 |
| 1NKL | 78 | 86 | 33 | 4.60 |
| 1H2Q | 125 | 47 | 18 | 4.61 |
| 3TGF | 50 | 34 | 13 | 4.61 |
| 1OMU | 56 | 34 | 13 | 4.61 |
| 1XAK | 83 | 34 | 13 | 4.61 |
| 4RSK | 124 | 76 | 29 | 4.61 |
| 1ML8 | 134 | 118 | 45 | 4.61 |
| 1BIG | 37 | 21 | 8 | 4.61 |
| 2BMT | 37 | 21 | 8 | 4.61 |
| 1X6Q | 123 | 84 | 32 | 4.61 |
| 1HP8 | 68 | 71 | 27 | 4.62 |
| 2HQI | 72 | 50 | 19 | 4.62 |
| 1B1U | 122 | 79 | 30 | 4.62 |
| 2JGW | 61 | 29 | 11 | 4.62 |
| 2BP2 | 130 | 95 | 36 | 4.62 |
| 2YX8 | 93 | 90 | 34 | 4.63 |
| 1E5C | 87 | 45 | 17 | 4.63 |
| 3SSI | 113 | 69 | 26 | 4.63 |
| 1RJH | 118 | 77 | 29 | 4.63 |
| 2QHE | 122 | 101 | 38 | 4.63 |
| 3EO5 | 171 | 125 | 47 | 4.63 |
| 1V6R | 21 | 8 | 3 | 4.64 |
| 1NIX | 33 | 8 | 3 | 4.64 |
| 1BK8 | 50 | 32 | 12 | 4.64 |
| 1LSI | 66 | 24 | 9 | 4.64 |
| 1QGM | 30 | 8 | 3 | 4.64 |
| 1CDQ | 77 | 32 | 12 | 4.64 |
| 6RAT | 124 | 80 | 30 | 4.64 |
| 1LY2 | 130 | 48 | 18 | 4.64 |
| 2FEB | 102 | 24 | 9 | 4.64 |
| 1EIH | 73 | 32 | 12 | 4.64 |
| 1IE5 | 107 | 56 | 21 | 4.64 |
| 3D4M | 109 | 112 | 42 | 4.64 |
| 2F3L | 184 | 102 | 38 | 4.65 |
| 1WKT | 88 | 43 | 16 | 4.65 |
| 2OVO | 56 | 35 | 13 | 4.65 |
| 4AIT | 74 | 35 | 13 | 4.65 |
| 1BVM | 123 | 97 | 36 | 4.65 |
| 2JP0 | 131 | 62 | 23 | 4.65 |
| 1B3C | 64 | 27 | 10 | 4.65 |
| 1Y1B | 48 | 27 | 10 | 4.65 |
| 1SJU | 50 | 27 | 10 | 4.65 |
| 2K51 | 77 | 27 | 10 | 4.65 |
| 1Z5F | 105 | 54 | 20 | 4.65 |
| 1RCK | 106 | 54 | 20 | 4.65 |
| 1MH8 | 119 | 100 | 37 | 4.65 |
| 1KNL | 130 | 73 | 27 | 4.66 |
| 1FOV | 82 | 73 | 27 | 4.66 |
| 2RNG | 79 | 46 | 17 | 4.66 |
| 1UOT | 125 | 46 | 17 | 4.66 |
| 1FUS | 106 | 65 | 24 | 4.66 |
| 1BUU | 168 | 130 | 48 | 4.66 |
| 1X6X | 123 | 84 | 31 | 4.66 |
| 9RAT | 124 | 76 | 28 | 4.66 |
| 1JFN | 119 | 38 | 14 | 4.66 |
| 1C5A | 73 | 68 | 25 | 4.66 |
| 1URK | 130 | 49 | 18 | 4.66 |
| 1VLK | 145 | 158 | 58 | 4.67 |
| 1CXO | 60 | 30 | 11 | 4.67 |
| 2JGX | 61 | 30 | 11 | 4.67 |
| 1WAQ | 117 | 60 | 22 | 4.67 |
| 1BQS | 209 | 90 | 33 | 4.67 |
| 2GF1 | 70 | 41 | 15 | 4.67 |
| 1B8K | 119 | 41 | 15 | 4.67 |
| 2EO9 | 118 | 41 | 15 | 4.67 |
| 1F32 | 149 | 104 | 38 | 4.67 |
| 2JZJ | 124 | 63 | 23 | 4.67 |
| 2UZR | 124 | 85 | 31 | 4.67 |
| 1S2B | 206 | 118 | 43 | 4.67 |
| 1ZYV | 66 | 33 | 12 | 4.68 |
| 1HSW | 129 | 99 | 36 | 4.68 |
| 2PF1 | 156 | 44 | 16 | 4.68 |
| 1UN3 | 123 | 77 | 28 | 4.68 |
| 2D49 | 53 | 22 | 8 | 4.68 |
| 2ENS | 96 | 33 | 12 | 4.68 |
| 1TVS | 75 | 47 | 17 | 4.68 |
| 1LR2 | 207 | 119 | 43 | 4.69 |
| 3DIH | 122 | 97 | 35 | 4.69 |
| 1BW3 | 125 | 61 | 22 | 4.69 |
| 1HK5 | 585 | 630 | 227 | 4.69 |
| 1IRH | 61 | 25 | 9 | 4.69 |
| 1E8R | 50 | 25 | 9 | 4.69 |
| 1E8E | 124 | 75 | 27 | 4.69 |
| 1LSF | 129 | 103 | 37 | 4.69 |
| 1R1M | 164 | 142 | 51 | 4.69 |
| 1HFH | 120 | 39 | 14 | 4.69 |
| 2AIT | 74 | 39 | 14 | 4.69 |
| 2RP3 | 101 | 53 | 19 | 4.70 |
| 1IEN | 19 | 14 | 5 | 4.70 |
| 1V7F | 29 | 14 | 5 | 4.70 |
| 1C4E | 35 | 14 | 5 | 4.70 |
| 2JOT | 55 | 28 | 10 | 4.70 |
| 1SHP | 55 | 28 | 10 | 4.70 |
| 1HPK | 79 | 28 | 10 | 4.70 |
| 1F2G | 58 | 28 | 10 | 4.70 |
| 1HEH | 88 | 28 | 10 | 4.70 |
| 1CFE | 135 | 101 | 36 | 4.70 |
| 1FLY | 129 | 107 | 38 | 4.71 |
| 1IJC | 63 | 31 | 11 | 4.71 |
| 1KDU | 85 | 31 | 11 | 4.71 |
| 2G0K | 122 | 62 | 22 | 4.71 |
| 1UIE | 129 | 110 | 39 | 4.71 |
| 1RHB | 124 | 79 | 28 | 4.71 |
| 5RAT | 124 | 79 | 28 | 4.71 |
| 1IYY | 104 | 48 | 17 | 4.71 |
| 2CR3 | 99 | 48 | 17 | 4.71 |
| 4RAT | 124 | 82 | 29 | 4.71 |
| 1X6Z | 123 | 82 | 29 | 4.71 |
| 1LR3 | 207 | 116 | 41 | 4.71 |
| 1KWN | 207 | 116 | 41 | 4.71 |
| 2BDS | 43 | 17 | 6 | 4.72 |
| 2DDJ | 70 | 34 | 12 | 4.72 |
| 1UIB | 127 | 102 | 36 | 4.72 |
| 4LYM | 129 | 102 | 36 | 4.72 |
| 1QUB | 319 | 139 | 49 | 4.72 |
| 2GTG | 83 | 88 | 31 | 4.72 |
| 1OF9 | 77 | 71 | 25 | 4.72 |
| 1ZLB | 122 | 108 | 38 | 4.72 |
| 3LZ2 | 129 | 91 | 32 | 4.72 |
| 1G4G | 86 | 37 | 13 | 4.72 |
| 1B2T | 77 | 37 | 13 | 4.72 |
| 1RBX | 124 | 77 | 27 | 4.72 |
| 1E8P | 46 | 20 | 7 | 4.73 |
| 1UUC | 55 | 40 | 14 | 4.73 |
| 3AIT | 74 | 40 | 14 | 4.73 |
| 1Q3M | 49 | 20 | 7 | 4.73 |
| 1J0T | 78 | 63 | 22 | 4.73 |
| 1RMS | 105 | 63 | 22 | 4.73 |
| 2YWZ | 111 | 63 | 22 | 4.73 |
| 1UIC | 129 | 106 | 37 | 4.73 |
| 1B0D | 129 | 106 | 37 | 4.73 |
| 1LZG | 129 | 106 | 37 | 4.73 |
| 1O9X | 585 | 576 | 201 | 4.73 |
| 2OP2 | 124 | 86 | 30 | 4.73 |
| 1JIT | 129 | 109 | 38 | 4.73 |
| 1INR | 160 | 155 | 54 | 4.73 |
| 1FN5 | 129 | 112 | 39 | 4.73 |
| 1CVO | 62 | 23 | 8 | 4.74 |
| 1UUA | 56 | 23 | 8 | 4.74 |
| 1EXG | 110 | 69 | 24 | 4.74 |
| 1ZNY | 207 | 164 | 57 | 4.74 |
| 1C3Z | 108 | 72 | 25 | 4.74 |
| 1OWT | 66 | 49 | 17 | 4.74 |
| 1TGJ | 112 | 75 | 26 | 4.74 |
| 6RSA | 124 | 75 | 26 | 4.74 |
| 1E7F | 585 | 612 | 212 | 4.74 |
| 1BTI | 58 | 26 | 9 | 4.74 |
| 1ROB | 124 | 78 | 27 | 4.74 |
| 1UIA | 127 | 104 | 36 | 4.74 |
| 1XFL | 124 | 104 | 36 | 4.74 |
| 1RAT | 124 | 81 | 28 | 4.74 |
| 3RAT | 124 | 81 | 28 | 4.74 |
| 7RAT | 124 | 81 | 28 | 4.74 |
| 1AKI | 129 | 110 | 38 | 4.74 |
| 1BWJ | 129 | 110 | 38 | 4.74 |
| 4RNT | 104 | 55 | 19 | 4.74 |
| 1EIC | 124 | 84 | 29 | 4.74 |
| 1XEK | 129 | 84 | 29 | 4.74 |
| 1ZNW | 207 | 168 | 58 | 4.74 |
| 1LZY | 129 | 113 | 39 | 4.74 |
| 8PTI | 58 | 29 | 10 | 4.75 |
| 1UVF | 61 | 29 | 10 | 4.75 |
| 2YVB | 129 | 116 | 40 | 4.75 |
| 1DQC | 73 | 32 | 11 | 4.75 |
| 1KJS | 74 | 64 | 22 | 4.75 |
| 1ZNX | 207 | 160 | 55 | 4.75 |
| 2O6R | 177 | 99 | 34 | 4.75 |
| 1LSD | 129 | 105 | 36 | 4.75 |
| 1UIH | 129 | 108 | 37 | 4.75 |
| 1MZD | 240 | 108 | 37 | 4.75 |
| 1IR7 | 129 | 111 | 38 | 4.76 |
| 1RHA | 124 | 76 | 26 | 4.76 |
| 1EID | 124 | 79 | 27 | 4.76 |
| 1E5B | 87 | 41 | 14 | 4.76 |
| 2BXL | 585 | 637 | 217 | 4.76 |
| 2AF9 | 164 | 91 | 31 | 4.76 |
| 2BXQ | 585 | 652 | 222 | 4.76 |
| 1XFT | 129 | 94 | 32 | 4.76 |
| 2OV8 | 288 | 191 | 65 | 4.76 |
| 2BXK | 585 | 641 | 218 | 4.76 |
| 1WQ8 | 110 | 50 | 17 | 4.76 |
| 1IA5 | 339 | 253 | 86 | 4.76 |
| 1HEQ | 129 | 109 | 37 | 4.77 |
| 1UIF | 129 | 112 | 38 | 4.77 |
| 1LY0 | 207 | 118 | 40 | 4.77 |
| 5RNT | 104 | 62 | 21 | 4.77 |
| 1FUT | 106 | 62 | 21 | 4.77 |
| 2RHE | 114 | 62 | 21 | 4.77 |
| 2K11 | 127 | 65 | 22 | 4.77 |
| 1XFR | 128 | 139 | 47 | 4.77 |
| 1C3Y | 108 | 71 | 24 | 4.77 |
| 2R1Q | 85 | 74 | 25 | 4.77 |
| 1N3Z | 124 | 74 | 25 | 4.77 |
| 7RSA | 124 | 77 | 26 | 4.77 |
| 2QCA | 124 | 77 | 26 | 4.77 |
| 1B1J | 123 | 77 | 26 | 4.77 |
| 1UN4 | 123 | 77 | 26 | 4.77 |
| 1TRS | 105 | 80 | 27 | 4.77 |
| 1BB6 | 129 | 95 | 32 | 4.78 |
| 1MG6 | 122 | 98 | 33 | 4.78 |
| 1E7G | 585 | 603 | 203 | 4.78 |
| 1LZB | 129 | 110 | 37 | 4.78 |
| 1LSZ | 147 | 110 | 37 | 4.78 |
| 2BLU | 206 | 116 | 39 | 4.78 |
| 2BLR | 206 | 116 | 39 | 4.78 |
| 2G4Y | 207 | 122 | 41 | 4.78 |
| 1TRY | 224 | 122 | 41 | 4.78 |
| 2A91 | 517 | 271 | 91 | 4.78 |
| 1BJ5 | 585 | 582 | 195 | 4.78 |
| 1BKE | 581 | 589 | 197 | 4.78 |
| 2K9E | 37 | 18 | 6 | 4.79 |
| 1ZFI | 67 | 30 | 10 | 4.79 |
| 1KS6 | 107 | 45 | 15 | 4.79 |
| 1KUN | 58 | 27 | 9 | 4.79 |
| 2HCC | 66 | 33 | 11 | 4.79 |
| 1Z3Q | 200 | 108 | 36 | 4.79 |
| 1LFC | 25 | 6 | 2 | 4.79 |
| 1S6D | 103 | 48 | 16 | 4.79 |
| 2OQN | 207 | 120 | 40 | 4.79 |
| 1LXZ | 207 | 117 | 39 | 4.79 |
| 2PK4 | 80 | 27 | 9 | 4.79 |
| 1BFA | 134 | 81 | 27 | 4.79 |
| 2BHK | 120 | 66 | 22 | 4.79 |
| 1RNQ | 124 | 75 | 25 | 4.79 |
| 1KS0 | 63 | 27 | 9 | 4.79 |
| 1GKN | 128 | 45 | 15 | 4.79 |
| 1F10 | 129 | 111 | 37 | 4.79 |
| 2LYM | 129 | 111 | 37 | 4.79 |
| 1LZE | 129 | 105 | 35 | 4.79 |
| 1XEJ | 129 | 93 | 31 | 4.79 |
| 1NWV | 129 | 45 | 15 | 4.79 |
| 2ERS | 66 | 27 | 9 | 4.79 |
| 2JON | 101 | 51 | 17 | 4.79 |
| 3BFA | 119 | 117 | 39 | 4.79 |
| 3CAB | 119 | 117 | 39 | 4.79 |
| 1ANG | 123 | 81 | 27 | 4.79 |
| 1RNT | 104 | 63 | 21 | 4.79 |
| 1BOX | 96 | 54 | 18 | 4.79 |
| 1T2I | 96 | 54 | 18 | 4.79 |
| 1BAJ | 101 | 69 | 23 | 4.79 |
| 1WYJ | 125 | 48 | 16 | 4.79 |
| 1O7V | 127 | 69 | 23 | 4.79 |
| 3CTF | 129 | 108 | 36 | 4.79 |
| 2OVF | 288 | 213 | 71 | 4.79 |
| 1E7H | 585 | 633 | 210 | 4.79 |
| 3BJH | 119 | 118 | 39 | 4.80 |
| 1JSE | 129 | 115 | 38 | 4.80 |
| 2C8P | 129 | 115 | 38 | 4.80 |
| 1LHJ | 130 | 115 | 38 | 4.80 |
| 1FLQ | 129 | 112 | 37 | 4.80 |
| 1JJ0 | 129 | 112 | 37 | 4.80 |
| 1KXW | 129 | 112 | 37 | 4.80 |
| 1IOQ | 129 | 112 | 37 | 4.80 |
| 3LYM | 129 | 109 | 36 | 4.80 |
| 1H9Z | 585 | 639 | 211 | 4.80 |
| 1JIY | 129 | 106 | 35 | 4.80 |
| 1XEI | 129 | 103 | 34 | 4.80 |
| 1LSC | 129 | 103 | 34 | 4.80 |
| 1E7E | 585 | 603 | 199 | 4.80 |
| 1KTE | 105 | 100 | 33 | 4.80 |
| 2HG0 | 408 | 194 | 64 | 4.80 |
| 1IZR | 124 | 82 | 27 | 4.80 |
| 2RAT | 124 | 82 | 27 | 4.80 |
| 1IZP | 124 | 82 | 27 | 4.80 |
| 1RPG | 124 | 82 | 27 | 4.80 |
| 1TRU | 105 | 82 | 27 | 4.80 |
| 1KF5 | 124 | 79 | 26 | 4.80 |
| 1RBN | 124 | 79 | 26 | 4.80 |
| 1EIE | 124 | 79 | 26 | 4.80 |
| 1RBW | 124 | 76 | 25 | 4.80 |
| 8RAT | 124 | 76 | 25 | 4.80 |
| 1IY4 | 130 | 76 | 25 | 4.80 |
| 1UN5 | 125 | 76 | 25 | 4.80 |
| 1N8Y | 608 | 301 | 99 | 4.80 |
| 1KNM | 130 | 73 | 24 | 4.80 |
| 1UAP | 154 | 73 | 24 | 4.80 |
| 1JI8 | 111 | 70 | 23 | 4.81 |
| 1A43 | 87 | 67 | 22 | 4.81 |
| 2BXO | 585 | 646 | 212 | 4.81 |
| 2BXM | 585 | 643 | 211 | 4.81 |
| 1LXI | 139 | 64 | 21 | 4.81 |
| 1TGK | 112 | 61 | 20 | 4.81 |
| 2LVE | 114 | 61 | 20 | 4.81 |
| 1HEL | 129 | 113 | 37 | 4.81 |
| 2C8O | 129 | 113 | 37 | 4.81 |
| 1VED | 129 | 113 | 37 | 4.81 |
| 1JJ1 | 129 | 113 | 37 | 4.81 |
| 1HER | 129 | 110 | 36 | 4.81 |
| 1UIG | 129 | 110 | 36 | 4.81 |
| 1LHI | 130 | 110 | 36 | 4.81 |
| 1LSA | 129 | 107 | 35 | 4.81 |
| 1DKJ | 129 | 107 | 35 | 4.81 |
| 1IR9 | 129 | 107 | 35 | 4.81 |
| 1VDS | 129 | 107 | 35 | 4.81 |
| 1JPO | 129 | 107 | 35 | 4.81 |
| 3E3D | 129 | 107 | 35 | 4.81 |
| 1HK4 | 585 | 639 | 209 | 4.81 |
| 1LZC | 129 | 104 | 34 | 4.81 |
| 1KT3 | 183 | 104 | 34 | 4.81 |
| 2ZG1 | 214 | 101 | 33 | 4.81 |
| 2RLQ | 129 | 49 | 16 | 4.81 |
| 1LYZ | 129 | 95 | 31 | 4.81 |
| 1I56 | 130 | 92 | 30 | 4.81 |
| 1J9O | 93 | 46 | 15 | 4.81 |
| 1GNJ | 585 | 657 | 214 | 4.82 |
| 1TJD | 216 | 172 | 56 | 4.82 |
| 1RUV | 124 | 83 | 27 | 4.82 |
| 1E7C | 585 | 615 | 200 | 4.82 |
| 1AHM | 129 | 40 | 13 | 4.82 |
| 1RQW | 207 | 117 | 38 | 4.82 |
| 1KF7 | 124 | 77 | 25 | 4.82 |
| 1YMN | 124 | 77 | 25 | 4.82 |
| 2BVB | 137 | 77 | 25 | 4.82 |
| 2BXP | 585 | 653 | 212 | 4.82 |
| 1THW | 207 | 114 | 37 | 4.82 |
| 1C1Z | 326 | 151 | 49 | 4.82 |
| 1N1X | 124 | 74 | 24 | 4.82 |
| 1RSM | 124 | 74 | 24 | 4.82 |
| 1RFP | 129 | 111 | 36 | 4.82 |
| 1VDT | 129 | 111 | 36 | 4.82 |
| 1TVT | 75 | 37 | 12 | 4.82 |
| 2BXI | 585 | 660 | 214 | 4.82 |
| 1LSB | 129 | 108 | 35 | 4.82 |
| 1IOS | 129 | 108 | 35 | 4.82 |
| 1FLW | 129 | 108 | 35 | 4.82 |
| 1HSX | 129 | 105 | 34 | 4.82 |
| 1LMQ | 129 | 105 | 34 | 4.82 |
| 1TUJ | 123 | 99 | 32 | 4.82 |
| 1TYN | 223 | 130 | 42 | 4.83 |
| 1LSE | 129 | 96 | 31 | 4.83 |
| 1LMN | 129 | 96 | 31 | 4.83 |
| 1B3J | 274 | 189 | 61 | 4.83 |
| 1HA2 | 585 | 654 | 211 | 4.83 |
| 1CXN | 60 | 31 | 10 | 4.83 |
| 1OA6 | 58 | 31 | 10 | 4.83 |
| 1REU | 103 | 62 | 20 | 4.83 |
| 1HFG | 71 | 31 | 10 | 4.83 |
| 1IKM | 72 | 31 | 10 | 4.83 |
| 1U53 | 196 | 155 | 50 | 4.83 |
| 1A67 | 108 | 62 | 20 | 4.83 |
| 2HRL | 127 | 62 | 20 | 4.83 |
| 4LYZ | 129 | 90 | 29 | 4.83 |
| 5LYZ | 129 | 90 | 29 | 4.83 |
| 1IVM | 130 | 90 | 29 | 4.83 |
| 2AHN | 222 | 118 | 38 | 4.83 |
| 1O7S | 127 | 59 | 19 | 4.83 |
| 1ILK | 151 | 171 | 55 | 4.83 |
| 1JIS | 129 | 112 | 36 | 4.83 |
| 1KXY | 129 | 112 | 36 | 4.83 |
| 135L | 129 | 109 | 35 | 4.83 |
| 1HEO | 129 | 109 | 35 | 4.83 |
| 2ZQ4 | 129 | 109 | 35 | 4.83 |
| 1LZA | 129 | 109 | 35 | 4.83 |
| 1RAS | 123 | 81 | 26 | 4.83 |
| 1YMW | 124 | 81 | 26 | 4.83 |
| 1IZQ | 124 | 81 | 26 | 4.83 |
| 1F0W | 129 | 106 | 34 | 4.83 |
| 1A8O | 70 | 53 | 17 | 4.83 |
| 1GV7 | 123 | 78 | 25 | 4.83 |
| 1YMR | 124 | 78 | 25 | 4.83 |
| 3RSD | 124 | 78 | 25 | 4.83 |
| 1KF8 | 124 | 78 | 25 | 4.83 |
| 2LYO | 129 | 103 | 33 | 4.83 |
| 2CRS | 60 | 25 | 8 | 4.84 |
| 1KBT | 60 | 25 | 8 | 4.84 |
| 1GU3 | 149 | 75 | 24 | 4.84 |
| 1E5U | 187 | 100 | 32 | 4.84 |
| 1Z8F | 228 | 169 | 54 | 4.84 |
| 1FY1 | 225 | 119 | 38 | 4.84 |
| 1I17 | 107 | 94 | 30 | 4.84 |
| 2JOB | 102 | 94 | 30 | 4.84 |
| 1HG8 | 349 | 251 | 80 | 4.84 |
| 132L | 129 | 91 | 29 | 4.84 |
| 1IOT | 129 | 113 | 36 | 4.84 |
| 1KBS | 60 | 22 | 7 | 4.84 |
| 1E8Q | 46 | 22 | 7 | 4.84 |
| 1BMP | 139 | 66 | 21 | 4.84 |
| 3BMP | 114 | 66 | 21 | 4.84 |
| 2H24 | 160 | 173 | 55 | 4.84 |
| 1LZD | 129 | 107 | 34 | 4.84 |
| 1LSY | 147 | 107 | 34 | 4.84 |
| 2HS9 | 129 | 85 | 27 | 4.84 |
| 1X91 | 153 | 211 | 67 | 4.85 |
| 1QO6 | 101 | 41 | 13 | 4.85 |
| 1EOT | 74 | 41 | 13 | 4.85 |
| 1DOL | 77 | 41 | 13 | 4.85 |
| 1J8I | 93 | 41 | 13 | 4.85 |
| 1Z3T | 431 | 265 | 84 | 4.85 |
| 1GNI | 585 | 651 | 206 | 4.85 |
| 1CHG | 245 | 136 | 43 | 4.85 |
| 1TPN | 50 | 19 | 6 | 4.85 |
| 1C01 | 76 | 38 | 12 | 4.85 |
| 1M58 | 106 | 57 | 18 | 4.85 |
| 1RPF | 124 | 76 | 24 | 4.85 |
| 1JYJ | 183 | 114 | 36 | 4.85 |
| 2I69 | 403 | 209 | 66 | 4.85 |
| 1ZYB | 232 | 190 | 60 | 4.85 |
| 1LVL | 458 | 358 | 113 | 4.85 |
| 1LSM | 129 | 111 | 35 | 4.85 |
| 2IIY | 105 | 92 | 29 | 4.85 |
| 1M9Z | 111 | 54 | 17 | 4.86 |
| 1VVD | 118 | 54 | 17 | 4.86 |
| 1VDQ | 129 | 108 | 34 | 4.86 |
| 1FLU | 129 | 108 | 34 | 4.86 |
| 1BWH | 129 | 108 | 34 | 4.86 |
| 1Z3W | 431 | 267 | 84 | 4.86 |
| 2BXN | 585 | 636 | 200 | 4.86 |
| 1ADZ | 71 | 35 | 11 | 4.86 |
| 1TFG | 112 | 70 | 22 | 4.86 |
| 2LZ2 | 129 | 86 | 27 | 4.86 |
| 1TWO | 142 | 99 | 31 | 4.86 |
| 1H0L | 112 | 99 | 31 | 4.86 |
| 1IK0 | 113 | 99 | 31 | 4.86 |
| 2I30 | 585 | 572 | 179 | 4.86 |
| 2FJZ | 59 | 48 | 15 | 4.86 |
| 2FMA | 59 | 48 | 15 | 4.86 |
| 1TPM | 50 | 16 | 5 | 4.86 |
| 1RNN | 124 | 80 | 25 | 4.86 |
| 1BWI | 129 | 112 | 35 | 4.86 |
| 1IOR | 129 | 112 | 35 | 4.86 |
| 1HEN | 129 | 112 | 35 | 4.86 |
| 1HEM | 129 | 112 | 35 | 4.86 |
| 1BOL | 222 | 160 | 50 | 4.86 |
| 1O7B | 98 | 64 | 20 | 4.86 |
| 2FBO | 250 | 128 | 40 | 4.86 |
| 2O85 | 107 | 80 | 25 | 4.86 |
| 1UC0 | 129 | 109 | 34 | 4.87 |
| 1RTB | 124 | 77 | 24 | 4.87 |
| 1BY2 | 119 | 61 | 19 | 4.87 |
| 1UOR | 585 | 424 | 132 | 4.87 |
| 2V44 | 189 | 106 | 33 | 4.87 |
| 2JTY | 184 | 106 | 33 | 4.87 |
| 1KI0 | 253 | 90 | 28 | 4.87 |
| 3D4T | 116 | 74 | 23 | 4.87 |
| 6LYT | 129 | 103 | 32 | 4.87 |
| 1HEW | 129 | 103 | 32 | 4.87 |
| 3LYO | 129 | 103 | 32 | 4.87 |
| 1RE2 | 130 | 103 | 32 | 4.87 |
| 1W8A | 192 | 103 | 32 | 4.87 |
| 1WFR | 143 | 103 | 32 | 4.87 |
| 1E7I | 585 | 596 | 185 | 4.87 |
| 2H0P | 112 | 58 | 18 | 4.87 |
| 2EGT | 132 | 116 | 36 | 4.87 |
| 1FAA | 124 | 100 | 31 | 4.87 |
| 3DFL | 263 | 142 | 44 | 4.87 |
| 3EXD | 129 | 113 | 35 | 4.87 |
| 1UID | 129 | 113 | 35 | 4.87 |
| 2BLX | 129 | 113 | 35 | 4.87 |
| 2P71 | 132 | 155 | 48 | 4.87 |
| 1KXX | 129 | 110 | 34 | 4.88 |
| 2EPE | 129 | 110 | 34 | 4.88 |
| 2BQI | 130 | 110 | 34 | 4.88 |
| 1Z3V | 431 | 275 | 85 | 4.88 |
| 2HI2 | 158 | 110 | 34 | 4.88 |
| 2TGI | 112 | 68 | 21 | 4.88 |
| 1GPI | 431 | 272 | 84 | 4.88 |
| 1NKO | 132 | 68 | 21 | 4.88 |
| 1BEA | 127 | 81 | 25 | 4.88 |
| 1E8B | 160 | 81 | 25 | 4.88 |
| 1RQM | 105 | 81 | 25 | 4.88 |
| 2CDS | 129 | 107 | 33 | 4.88 |
| 1AT6 | 129 | 107 | 33 | 4.88 |
| 3C1R | 118 | 107 | 33 | 4.88 |
| 1DIC | 228 | 120 | 37 | 4.88 |
| 2JPO | 142 | 133 | 41 | 4.88 |
| 1THV | 207 | 117 | 36 | 4.88 |
| 1BC4 | 111 | 65 | 20 | 4.88 |
| 1H1H | 134 | 78 | 24 | 4.88 |
| 3B6L | 147 | 104 | 32 | 4.88 |
| 1T6E | 381 | 208 | 64 | 4.88 |
| 2I85 | 142 | 65 | 20 | 4.88 |
| 1LSN | 129 | 114 | 35 | 4.88 |
| 2NWD | 130 | 114 | 35 | 4.88 |
| 1ITI | 133 | 114 | 35 | 4.88 |
| 1BHZ | 129 | 101 | 31 | 4.88 |
| 1E1J | 104 | 88 | 27 | 4.88 |
| 1EX3 | 245 | 150 | 46 | 4.88 |
| 2FRG | 106 | 62 | 19 | 4.89 |
| 1FZZ | 240 | 124 | 38 | 4.89 |
| 2AUB | 129 | 111 | 34 | 4.89 |
| 207L | 130 | 111 | 34 | 4.89 |
| 1PPA | 121 | 98 | 30 | 4.89 |
| 1BHU | 102 | 49 | 15 | 4.89 |
| 2RN4 | 106 | 49 | 15 | 4.89 |
| 1JJJ | 135 | 98 | 30 | 4.89 |
| 3BU9 | 148 | 85 | 26 | 4.89 |
| 1H46 | 431 | 278 | 85 | 4.89 |
| 1RC9 | 221 | 157 | 48 | 4.89 |
| 1HEP | 129 | 108 | 33 | 4.89 |
| 1BVX | 129 | 108 | 33 | 4.89 |
| 1SDF | 67 | 36 | 11 | 4.89 |
| 4ULL | 69 | 36 | 11 | 4.89 |
| 8LYZ | 129 | 95 | 29 | 4.89 |
| 1BBN | 133 | 118 | 36 | 4.89 |
| 1H75 | 81 | 59 | 18 | 4.89 |
| 2I2Z | 585 | 610 | 186 | 4.89 |
| 2GVS | 109 | 82 | 25 | 4.89 |
| 1KLX | 138 | 174 | 53 | 4.89 |
| 1VVC | 118 | 46 | 14 | 4.89 |
| 1C54 | 96 | 46 | 14 | 4.89 |
| 2SFA | 191 | 125 | 38 | 4.89 |
| 2AAS | 124 | 79 | 24 | 4.90 |
| 1JBJ | 186 | 79 | 24 | 4.90 |
| 2O7K | 107 | 79 | 24 | 4.90 |
| 1HNL | 130 | 112 | 34 | 4.90 |
| 1FP5 | 222 | 112 | 34 | 4.90 |
| 1HK3 | 585 | 593 | 180 | 4.90 |
| 2AGC | 162 | 89 | 27 | 4.90 |
| 1Y2S | 113 | 89 | 27 | 4.90 |
| 1LZT | 129 | 99 | 30 | 4.90 |
| 1A90 | 108 | 66 | 20 | 4.90 |
| 3B9M | 585 | 578 | 175 | 4.90 |
| 1LHL | 130 | 109 | 33 | 4.90 |
| 2JS9 | 99 | 76 | 23 | 4.90 |
| 1K5A | 123 | 76 | 23 | 4.90 |
| 2A7I | 207 | 119 | 36 | 4.90 |
| 1AHK | 129 | 43 | 13 | 4.90 |
| 1YO4 | 87 | 43 | 13 | 4.90 |
| 1P1N | 263 | 215 | 65 | 4.90 |
| 3LYZ | 129 | 96 | 29 | 4.90 |
| 1LMC | 129 | 96 | 29 | 4.90 |
| 1GSM | 210 | 96 | 29 | 4.90 |
| 1BR9 | 194 | 106 | 32 | 4.90 |
| 1N5H | 105 | 53 | 16 | 4.90 |
| 2ENG | 210 | 116 | 35 | 4.90 |
| 1IR8 | 129 | 116 | 35 | 4.90 |
| 2CL2 | 298 | 189 | 57 | 4.90 |
| 1AM5 | 324 | 189 | 57 | 4.90 |
| 1L6P | 125 | 63 | 19 | 4.90 |
| 1CUB | 214 | 156 | 47 | 4.90 |
| 2QO6 | 126 | 83 | 25 | 4.90 |
| 1LSG | 144 | 93 | 28 | 4.91 |
| 1K19 | 112 | 93 | 28 | 4.91 |
| 2FLS | 132 | 103 | 31 | 4.91 |
| 1MZA | 240 | 113 | 34 | 4.91 |
| 1C2U | 35 | 10 | 3 | 4.91 |
| 1OA5 | 58 | 30 | 9 | 4.91 |
| 2JSB | 21 | 10 | 3 | 4.91 |
| 1FS3 | 124 | 80 | 24 | 4.91 |
| 1LYY | 130 | 110 | 33 | 4.91 |
| 2JNC | 119 | 20 | 6 | 4.91 |
| 2BF1 | 316 | 110 | 33 | 4.91 |
| 1RDS | 105 | 60 | 18 | 4.91 |
| 2I83 | 160 | 60 | 18 | 4.91 |
| 1HFD | 228 | 120 | 36 | 4.91 |
| 1SVR | 114 | 70 | 21 | 4.91 |
| 1E0W | 313 | 267 | 80 | 4.91 |
| 1Q9H | 437 | 257 | 77 | 4.91 |
| 2SGA | 181 | 97 | 29 | 4.91 |
| 1QLZ | 210 | 97 | 29 | 4.91 |
| 1K4Q | 461 | 368 | 110 | 4.91 |
| 2GZZ | 104 | 87 | 26 | 4.91 |
| 3D0J | 140 | 77 | 23 | 4.91 |
| 1WDE | 294 | 231 | 69 | 4.91 |
| 1SVB | 395 | 211 | 63 | 4.91 |
| 1BIO | 228 | 124 | 37 | 4.91 |
| 2JZR | 144 | 104 | 31 | 4.92 |
| 1ERU | 105 | 94 | 28 | 4.92 |
| 1IJZ | 113 | 94 | 28 | 4.92 |
| 2CGY | 101 | 47 | 14 | 4.92 |
| 2JQM | 112 | 47 | 14 | 4.92 |
| 1UNP | 121 | 94 | 28 | 4.92 |
| 1FBR | 93 | 37 | 11 | 4.92 |
| 1N8U | 112 | 111 | 33 | 4.92 |
| 1PEE | 180 | 111 | 33 | 4.92 |
| 1EUO | 180 | 111 | 33 | 4.92 |
| 1PB7 | 292 | 222 | 66 | 4.92 |
| 4LYO | 129 | 101 | 30 | 4.92 |
| 1B7U | 689 | 479 | 142 | 4.92 |
| 4PTI | 58 | 27 | 8 | 4.92 |
| 1JV8 | 58 | 27 | 8 | 4.92 |
| 4KIV | 79 | 27 | 8 | 4.92 |
| 2J6D | 65 | 27 | 8 | 4.92 |
| 1HFN | 71 | 27 | 8 | 4.92 |
| 1MAJ | 113 | 54 | 16 | 4.92 |
| 1IRL | 133 | 108 | 32 | 4.92 |
| 2D9C | 136 | 54 | 16 | 4.92 |
| 1O73 | 144 | 108 | 32 | 4.92 |
| 2IEM | 211 | 125 | 37 | 4.92 |
| 1BB7 | 129 | 98 | 29 | 4.92 |
| 1BGD | 175 | 186 | 55 | 4.92 |
| 1JWR | 130 | 115 | 34 | 4.92 |
| 3BS2 | 148 | 88 | 26 | 4.93 |
| 1CDY | 178 | 105 | 31 | 4.93 |
| 1G84 | 105 | 61 | 18 | 4.93 |
| 1G9E | 117 | 61 | 18 | 4.93 |
| 2H9U | 102 | 78 | 23 | 4.93 |
| 2LYZ | 129 | 95 | 28 | 4.93 |
| 1THU | 207 | 112 | 33 | 4.93 |
| 2BLY | 129 | 112 | 33 | 4.93 |
| 1GOD | 121 | 85 | 25 | 4.93 |
| 1OUV | 273 | 357 | 105 | 4.93 |
| 1GZ2 | 142 | 85 | 25 | 4.93 |
| 1AH1 | 129 | 51 | 15 | 4.93 |
| 1TIN | 68 | 34 | 10 | 4.93 |
| 1TOF | 112 | 102 | 30 | 4.93 |
| 2QO4 | 126 | 85 | 25 | 4.93 |
| 1FW0 | 263 | 204 | 60 | 4.93 |
| 1BP1 | 456 | 340 | 100 | 4.93 |
| 1CKH | 130 | 109 | 32 | 4.93 |
| 1REZ | 130 | 109 | 32 | 4.93 |
| 2VA4 | 192 | 109 | 32 | 4.93 |
| 1FPV | 584 | 201 | 59 | 4.93 |
| 2C6J | 338 | 249 | 73 | 4.93 |
| 1DST | 228 | 116 | 34 | 4.93 |
| 2DF3 | 127 | 58 | 17 | 4.93 |
| 2PET | 231 | 116 | 34 | 4.93 |
| 1VMC | 71 | 41 | 12 | 4.94 |
| 1HE7 | 126 | 41 | 12 | 4.94 |
| 1D6P | 130 | 106 | 31 | 4.94 |
| 1RBP | 182 | 106 | 31 | 4.94 |
| 1T3B | 211 | 171 | 50 | 4.94 |
| 2GMT | 245 | 130 | 38 | 4.94 |
| 1LG4 | 129 | 65 | 19 | 4.94 |
| 1L5K | 356 | 332 | 97 | 4.94 |
| 1TGN | 229 | 113 | 33 | 4.94 |
| 3B9L | 585 | 593 | 173 | 4.94 |
| 1IIZ | 120 | 96 | 28 | 4.94 |
| 1GXX | 129 | 96 | 28 | 4.94 |
| 1SJX | 122 | 72 | 21 | 4.94 |
| 2FVN | 153 | 48 | 14 | 4.94 |
| 1HK1 | 585 | 611 | 178 | 4.94 |
| 2C9Y | 242 | 213 | 62 | 4.94 |
| 1TAY | 130 | 110 | 32 | 4.94 |
| 1I1Z | 130 | 110 | 32 | 4.94 |
| 1LZ4 | 130 | 110 | 32 | 4.94 |
| 1RK7 | 153 | 55 | 16 | 4.94 |
| 1A2J | 189 | 196 | 57 | 4.94 |
| 1DYM | 402 | 234 | 68 | 4.94 |
| 1DTV | 67 | 31 | 9 | 4.94 |
| 1DTK | 57 | 31 | 9 | 4.94 |
| 1KWI | 101 | 62 | 18 | 4.94 |
| 1CXY | 90 | 62 | 18 | 4.94 |
| 1LMO | 129 | 100 | 29 | 4.95 |
| 1AUM | 70 | 69 | 20 | 4.95 |
| 1HHL | 129 | 107 | 31 | 4.95 |
| 1NYO | 163 | 107 | 31 | 4.95 |
| 1PB9 | 292 | 221 | 64 | 4.95 |
| 1G4F | 86 | 38 | 11 | 4.95 |
| 1KT5 | 175 | 114 | 33 | 4.95 |
| 1JYD | 183 | 114 | 33 | 4.95 |
| 1OWJ | 245 | 121 | 35 | 4.95 |
| 2BOD | 286 | 242 | 70 | 4.95 |
| 1HJM | 104 | 83 | 24 | 4.95 |
| 2K74 | 183 | 166 | 48 | 4.95 |
| 1QOK | 282 | 128 | 37 | 4.95 |
| 2VAJ | 93 | 45 | 13 | 4.95 |
| 1SGC | 181 | 97 | 28 | 4.95 |
| 1BUY | 166 | 156 | 45 | 4.95 |
| 1LZ1 | 130 | 111 | 32 | 4.95 |
| 1C46 | 131 | 111 | 32 | 4.95 |
| 1LOZ | 130 | 118 | 34 | 4.95 |
| 1AZ8 | 223 | 118 | 34 | 4.95 |
| 1NP4 | 184 | 118 | 34 | 4.95 |
| 1MAK | 113 | 59 | 17 | 4.95 |
| 2BDL | 215 | 146 | 42 | 4.95 |
| 2EC8 | 524 | 226 | 65 | 4.95 |
| 1U9X | 217 | 153 | 44 | 4.95 |
| 1H52 | 123 | 80 | 23 | 4.95 |
| 1B1E | 123 | 80 | 23 | 4.95 |
| 1D0V | 356 | 334 | 96 | 4.95 |
| 134L | 130 | 108 | 31 | 4.96 |
| 1GM0 | 142 | 122 | 35 | 4.96 |
| 1FAZ | 122 | 143 | 41 | 4.96 |
| 1K36 | 46 | 14 | 4 | 4.96 |
| 1KSQ | 75 | 35 | 10 | 4.96 |
| 1HPJ | 79 | 28 | 8 | 4.96 |
| 4ENG | 210 | 119 | 34 | 4.96 |
| 3ENG | 213 | 119 | 34 | 4.96 |
| 1GXV | 129 | 98 | 28 | 4.96 |
| 1NTP | 223 | 119 | 34 | 4.96 |
| 2QXH | 224 | 126 | 36 | 4.96 |
| 2V5N | 299 | 126 | 36 | 4.96 |
| 1LWB | 122 | 140 | 40 | 4.96 |
| 1IT5 | 122 | 140 | 40 | 4.96 |
| 1EGO | 85 | 63 | 18 | 4.96 |
| 3CD4 | 182 | 98 | 28 | 4.96 |
| 2GV1 | 92 | 56 | 16 | 4.96 |
| 1P9A | 290 | 133 | 38 | 4.96 |
| 2O87 | 106 | 84 | 24 | 4.96 |
| 1R26 | 125 | 98 | 28 | 4.96 |
| 1B56 | 135 | 105 | 30 | 4.96 |
| 3BOA | 504 | 259 | 74 | 4.96 |
| 1LKE | 184 | 105 | 30 | 4.96 |
| 3DV8 | 220 | 175 | 50 | 4.96 |
| 1GRA | 478 | 378 | 108 | 4.96 |
| 1HK2 | 585 | 610 | 174 | 4.96 |
| 1N5U | 585 | 677 | 193 | 4.96 |
| 1L4M | 356 | 337 | 96 | 4.96 |
| 1AU4 | 215 | 144 | 41 | 4.96 |
| 2AMM | 180 | 116 | 33 | 4.97 |
| 1L5O | 356 | 341 | 97 | 4.97 |
| 1AUN | 208 | 109 | 31 | 4.97 |
| 1REX | 130 | 109 | 31 | 4.97 |
| 6LYZ | 129 | 95 | 27 | 4.97 |
| 2CE1 | 105 | 95 | 27 | 4.97 |
| 2LBP | 346 | 271 | 77 | 4.97 |
| 1HI4 | 135 | 88 | 25 | 4.97 |
| 1CUV | 214 | 169 | 48 | 4.97 |
| 1CEX | 214 | 162 | 46 | 4.97 |
| 2I4A | 107 | 81 | 23 | 4.97 |
| 1CEW | 108 | 74 | 21 | 4.97 |
| 1ABA | 87 | 74 | 21 | 4.97 |
| 1UH7 | 325 | 208 | 59 | 4.97 |
| 1T2J | 116 | 60 | 17 | 4.97 |
| 1UBZ | 130 | 113 | 32 | 4.97 |
| 1LHM | 130 | 113 | 32 | 4.97 |
| 1PM1 | 180 | 113 | 32 | 4.97 |
| 2BZM | 129 | 53 | 15 | 4.97 |
| 1JBI | 100 | 53 | 15 | 4.97 |
| 1DWY | 112 | 99 | 28 | 4.97 |
| 1QM0 | 143 | 92 | 26 | 4.97 |
| 2C7Z | 404 | 308 | 87 | 4.97 |
| 1GR2 | 279 | 209 | 59 | 4.97 |
| 1GVL | 223 | 124 | 35 | 4.97 |
| 1XZG | 214 | 163 | 46 | 4.97 |
| 2GO0 | 137 | 78 | 22 | 4.97 |
| 2E5E | 101 | 39 | 11 | 4.97 |
| 1TG8 | 395 | 213 | 60 | 4.98 |
| 1RFS | 139 | 71 | 20 | 4.98 |
| 1KNG | 156 | 103 | 29 | 4.98 |
| 1OWK | 245 | 135 | 38 | 4.98 |
| 1BHE | 376 | 263 | 74 | 4.98 |
| 1GD6 | 119 | 96 | 27 | 4.98 |
| 2ALA | 391 | 160 | 45 | 4.98 |
| 2V07 | 105 | 96 | 27 | 4.98 |
| 1BV8 | 138 | 64 | 18 | 4.98 |
| 1JHQ | 356 | 338 | 95 | 4.98 |
| 1HD5 | 213 | 121 | 34 | 4.98 |
| 1FTK | 279 | 210 | 59 | 4.98 |
| 2P70 | 132 | 146 | 41 | 4.98 |
| 2NVK | 488 | 374 | 105 | 4.98 |
| 1ZKQ | 517 | 374 | 105 | 4.98 |
| 1L6U | 128 | 57 | 16 | 4.98 |
| 2Z8H | 138 | 114 | 32 | 4.98 |
| 1T0V | 184 | 82 | 23 | 4.98 |
| 2BOE | 286 | 246 | 69 | 4.98 |
| 1LHH | 130 | 107 | 30 | 4.98 |
| 1TDY | 130 | 107 | 30 | 4.98 |
| 1BRQ | 182 | 107 | 30 | 4.98 |
| 1LKI | 180 | 182 | 51 | 4.98 |
| 2FN2 | 59 | 25 | 7 | 4.98 |
| 2JSA | 99 | 75 | 21 | 4.98 |
| 2HDL | 78 | 50 | 14 | 4.98 |
| 2JCR | 154 | 100 | 28 | 4.98 |
| 1WG1 | 88 | 50 | 14 | 4.98 |
| 3EOW | 221 | 75 | 21 | 4.98 |
| 2JV6 | 112 | 50 | 14 | 4.98 |
| 1JHA | 356 | 343 | 96 | 4.98 |
| 2AT6 | 184 | 118 | 33 | 4.98 |
| 1XZD | 214 | 161 | 45 | 4.98 |
| 1XZC | 214 | 161 | 45 | 4.98 |
| 1BED | 181 | 161 | 45 | 4.98 |
| 1E21 | 128 | 68 | 19 | 4.98 |
| 1T8C | 143 | 68 | 19 | 4.98 |
| 2OM5 | 381 | 179 | 50 | 4.98 |
| 2AH7 | 180 | 111 | 31 | 4.98 |
| 1U9W | 217 | 154 | 43 | 4.98 |
| 1HI5 | 135 | 86 | 24 | 4.99 |
| 1ZDC | 34 | 43 | 12 | 4.99 |
| 3IL8 | 72 | 43 | 12 | 4.99 |
| 1ESR | 76 | 43 | 12 | 4.99 |
| 2ZE4 | 509 | 405 | 113 | 4.99 |
| 1L4E | 356 | 337 | 94 | 4.99 |
| 1XZA | 214 | 165 | 46 | 4.99 |
| 1L8F | 207 | 122 | 34 | 4.99 |
| 1K59 | 123 | 79 | 22 | 4.99 |
| 2I1U | 121 | 79 | 22 | 4.99 |
| 1I6B | 689 | 413 | 115 | 4.99 |
| 2Z62 | 276 | 169 | 47 | 4.99 |
| 1BDS | 43 | 18 | 5 | 4.99 |
| 1B6E | 128 | 72 | 20 | 4.99 |
| 2BZZ | 135 | 90 | 25 | 4.99 |
| 208L | 130 | 108 | 30 | 4.99 |
| 2CV3 | 240 | 144 | 40 | 4.99 |
| 1E1U | 104 | 90 | 25 | 4.99 |
| 1CUJ | 214 | 162 | 45 | 4.99 |
| 1DX0 | 219 | 90 | 25 | 4.99 |
| 1L4H | 356 | 339 | 94 | 4.99 |
| 1MD6 | 154 | 83 | 23 | 4.99 |
| 2GMK | 104 | 65 | 18 | 4.99 |
| 1EVS | 187 | 195 | 54 | 4.99 |
| 2BQH | 130 | 112 | 31 | 4.99 |
| 1HBQ | 183 | 112 | 31 | 4.99 |
| 2A3F | 180 | 112 | 31 | 4.99 |
| 1QDD | 144 | 94 | 26 | 4.99 |
| 1IAM | 185 | 94 | 26 | 4.99 |
| 2GG1 | 102 | 47 | 13 | 4.99 |
| 2XYL | 312 | 286 | 79 | 5.00 |
| 1LAA | 130 | 105 | 29 | 5.00 |
| 1VSN | 215 | 134 | 37 | 5.00 |
| 1CUA | 214 | 163 | 45 | 5.00 |
| 1A39 | 402 | 250 | 69 | 5.00 |
| 2AYU | 417 | 250 | 69 | 5.00 |
| 1DEN | 60 | 29 | 8 | 5.00 |
| 3KIV | 79 | 29 | 8 | 5.00 |
| 2CA7 | 60 | 29 | 8 | 5.00 |
| 1IO5 | 129 | 87 | 24 | 5.00 |
| 2G5R | 127 | 58 | 16 | 5.00 |
| 1CC7 | 73 | 58 | 16 | 5.00 |
| 2CE0 | 105 | 98 | 27 | 5.00 |
| 1T8D | 143 | 69 | 19 | 5.00 |
| 1EXP | 312 | 287 | 79 | 5.00 |
| 133L | 130 | 109 | 30 | 5.00 |
| 1LF7 | 182 | 109 | 30 | 5.00 |
| 1YWC | 184 | 120 | 33 | 5.00 |
| 1CUE | 197 | 160 | 44 | 5.00 |
| 1P3C | 215 | 131 | 36 | 5.00 |
| 1DTZ | 689 | 484 | 133 | 5.00 |
| 2QEH | 145 | 142 | 39 | 5.00 |
| 1LNM | 184 | 102 | 28 | 5.00 |
| 1CUS | 200 | 164 | 45 | 5.00 |
| 1CYL | 129 | 113 | 31 | 5.00 |
| 2ACP | 180 | 113 | 31 | 5.00 |
| 2EXO | 312 | 288 | 79 | 5.00 |
| 1KVZ | 107 | 62 | 17 | 5.00 |
| 1ZKZ | 110 | 62 | 17 | 5.00 |
| 1ERX | 184 | 124 | 34 | 5.00 |
| 1P8T | 285 | 146 | 40 | 5.00 |
| 1WBC | 183 | 73 | 20 | 5.00 |
| 1K5B | 120 | 84 | 23 | 5.00 |
| 2CQ9 | 130 | 84 | 23 | 5.00 |
| 2EQL | 129 | 106 | 29 | 5.01 |
| 2LHM | 130 | 106 | 29 | 5.01 |
| 1REY | 130 | 106 | 29 | 5.01 |
| 1OA9 | 214 | 117 | 32 | 5.01 |
| 2ZB5 | 481 | 234 | 64 | 5.01 |
| 2JU5 | 154 | 117 | 32 | 5.01 |
| 2GV6 | 241 | 128 | 35 | 5.01 |
| 1AIV | 686 | 417 | 114 | 5.01 |
| 1AU3 | 215 | 150 | 41 | 5.01 |
| 1FFE | 214 | 161 | 44 | 5.01 |
| 1JHO | 356 | 344 | 94 | 5.01 |
| 1L5M | 356 | 337 | 92 | 5.01 |
| 1OJT | 482 | 381 | 104 | 5.01 |
| 1MKC | 43 | 11 | 3 | 5.01 |
| 1ICO | 29 | 11 | 3 | 5.01 |
| 1O8R | 94 | 44 | 12 | 5.01 |
| 7LYZ | 129 | 88 | 24 | 5.01 |
| 1D6Q | 130 | 110 | 30 | 5.01 |
| 2C05 | 135 | 88 | 24 | 5.01 |
| 1OOI | 124 | 132 | 36 | 5.01 |
| 2BQM | 130 | 110 | 30 | 5.01 |
| 1E1W | 104 | 99 | 27 | 5.01 |
| 1SJV | 114 | 44 | 12 | 5.01 |
| 1O4W | 147 | 110 | 30 | 5.01 |
| 1Y1Z | 292 | 224 | 61 | 5.01 |
| 1PB8 | 292 | 224 | 61 | 5.01 |
| 2CAS | 548 | 213 | 58 | 5.01 |
| 1PSN | 326 | 202 | 55 | 5.01 |
| 1AGY | 200 | 158 | 43 | 5.01 |
| 1XZE | 214 | 158 | 43 | 5.01 |
| 1U9V | 217 | 147 | 40 | 5.01 |
| 2IRM | 358 | 283 | 77 | 5.01 |
| 2QEV | 145 | 136 | 37 | 5.01 |
| 1ZYO | 191 | 114 | 31 | 5.01 |
| 1L5L | 356 | 342 | 93 | 5.01 |
| 2HZR | 174 | 92 | 25 | 5.01 |
| 1CUI | 214 | 162 | 44 | 5.01 |
| 1XSF | 108 | 81 | 22 | 5.01 |
| 2V7S | 215 | 151 | 41 | 5.01 |
| 2DKV | 309 | 221 | 60 | 5.01 |
| 1DE3 | 150 | 70 | 19 | 5.01 |
| 1XNC | 185 | 129 | 35 | 5.01 |
| 2APV | 112 | 59 | 16 | 5.01 |
| 1XZH | 214 | 166 | 45 | 5.01 |
| 1LHK | 130 | 107 | 29 | 5.01 |
| 1I20 | 130 | 107 | 29 | 5.01 |
| 1USG | 346 | 321 | 87 | 5.01 |
| 2BOF | 286 | 240 | 65 | 5.02 |
| 1XAN | 461 | 373 | 101 | 5.02 |
| 1E0V | 313 | 277 | 75 | 5.02 |
| 1CDJ | 178 | 85 | 23 | 5.02 |
| 2BOG | 286 | 244 | 66 | 5.02 |
| 1QZ1 | 291 | 159 | 43 | 5.02 |
| 1BHY | 482 | 381 | 103 | 5.02 |
| 2EOT | 74 | 37 | 10 | 5.02 |
| 1DWM | 69 | 37 | 10 | 5.02 |
| 1XWE | 151 | 74 | 20 | 5.02 |
| 2FR2 | 172 | 111 | 30 | 5.02 |
| 1L4L | 356 | 337 | 91 | 5.02 |
| 1HKF | 122 | 63 | 17 | 5.02 |
| 1HCV | 117 | 63 | 17 | 5.02 |
| 2O6Q | 270 | 152 | 41 | 5.02 |
| 1ESD | 306 | 241 | 65 | 5.02 |
| 2C01 | 135 | 89 | 24 | 5.02 |
| 2C02 | 135 | 89 | 24 | 5.02 |
| 2WBC | 183 | 89 | 24 | 5.02 |
| 2E8C | 132 | 115 | 31 | 5.02 |
| 1DEM | 60 | 26 | 7 | 5.02 |
| 2ZG3 | 214 | 104 | 28 | 5.02 |
| 2NCM | 99 | 52 | 14 | 5.02 |
| 1C8E | 548 | 208 | 56 | 5.02 |
| 2HZQ | 174 | 93 | 25 | 5.02 |
| 1PU3 | 105 | 67 | 18 | 5.02 |
| 1KT6 | 183 | 108 | 29 | 5.02 |
| 1KT4 | 183 | 108 | 29 | 5.02 |
| 1AE5 | 225 | 123 | 33 | 5.02 |
| 3PEP | 326 | 205 | 55 | 5.02 |
| 1ZX9 | 467 | 384 | 103 | 5.02 |
| 1L4G | 356 | 332 | 89 | 5.03 |
| 1UH9 | 325 | 209 | 56 | 5.03 |
| 2JOP | 131 | 56 | 15 | 5.03 |
| 2BQK | 130 | 112 | 30 | 5.03 |
| 2ALL | 180 | 112 | 30 | 5.03 |
| 1L4F | 356 | 336 | 90 | 5.03 |
| 2H6O | 470 | 183 | 49 | 5.03 |
| 1CWV | 492 | 258 | 69 | 5.03 |
| 1P3E | 215 | 131 | 35 | 5.03 |
| 1OZN | 285 | 161 | 43 | 5.03 |
| 1CUF | 214 | 161 | 43 | 5.03 |
| 1XZM | 214 | 161 | 43 | 5.03 |
| 1EWF | 456 | 341 | 91 | 5.03 |
| 1LD5 | 58 | 30 | 8 | 5.03 |
| 1E88 | 160 | 75 | 20 | 5.03 |
| 1QMT | 134 | 75 | 20 | 5.03 |
| 1JE4 | 69 | 30 | 8 | 5.03 |
| 1FGP | 70 | 45 | 12 | 5.03 |
| 2E56 | 144 | 75 | 20 | 5.03 |
| 1BGC | 174 | 195 | 52 | 5.03 |
| 1X8N | 184 | 120 | 32 | 5.03 |
| 1M7T | 107 | 75 | 20 | 5.03 |
| 1JPC | 109 | 60 | 16 | 5.03 |
| 1BEC | 238 | 120 | 32 | 5.03 |
| 1LVE | 122 | 60 | 16 | 5.03 |
| 1ESC | 306 | 244 | 65 | 5.03 |
| 1FLH | 326 | 199 | 53 | 5.03 |
| 1AQ7 | 223 | 124 | 33 | 5.03 |
| 2CUT | 198 | 173 | 46 | 5.03 |
| 1B1I | 123 | 79 | 21 | 5.03 |
| 1WY6 | 172 | 207 | 55 | 5.03 |
| 3E16 | 271 | 128 | 34 | 5.03 |
| 1QSV | 101 | 64 | 17 | 5.03 |
| 1ODA | 119 | 64 | 17 | 5.03 |
| 1PVX | 194 | 128 | 34 | 5.03 |
| 1Y20 | 292 | 226 | 60 | 5.03 |
| 1VB2 | 203 | 113 | 30 | 5.03 |
| 1XZF | 214 | 162 | 43 | 5.03 |
| 2JCQ | 154 | 98 | 26 | 5.04 |
| 1CDH | 178 | 98 | 26 | 5.04 |
| 3GRT | 461 | 362 | 96 | 5.04 |
| 3E0P | 271 | 132 | 35 | 5.04 |
| 1CUH | 214 | 166 | 44 | 5.04 |
| 1YWD | 184 | 117 | 31 | 5.04 |
| 2R6N | 217 | 151 | 40 | 5.04 |
| 1JHR | 356 | 336 | 89 | 5.04 |
| 2FX5 | 258 | 185 | 49 | 5.04 |
| 1G2T | 71 | 34 | 9 | 5.04 |
| 1GP3 | 143 | 68 | 18 | 5.04 |
| 1IKL | 72 | 34 | 9 | 5.04 |
| 1OWD | 245 | 136 | 36 | 5.04 |
| 2FWG | 134 | 102 | 27 | 5.04 |
| 1HI3 | 135 | 87 | 23 | 5.04 |
| 1J87 | 172 | 87 | 23 | 5.04 |
| 1E1G | 104 | 87 | 23 | 5.04 |
| 1QNO | 344 | 280 | 74 | 5.04 |
| 1XZJ | 214 | 159 | 42 | 5.04 |
| 1XZI | 214 | 159 | 42 | 5.04 |
| 1O85 | 146 | 106 | 28 | 5.04 |
| 2QXI | 224 | 125 | 33 | 5.04 |
| 2Q2P | 162 | 91 | 24 | 5.04 |
| 3APP | 323 | 201 | 53 | 5.04 |
| 2ASN | 179 | 110 | 29 | 5.04 |
| 1OJI | 402 | 239 | 63 | 5.04 |
| 1V0K | 313 | 281 | 74 | 5.04 |
| 5GRT | 461 | 357 | 94 | 5.04 |
| 1HA6 | 70 | 38 | 10 | 5.04 |
| 1B0F | 218 | 114 | 30 | 5.04 |
| 2AL0 | 180 | 114 | 30 | 5.04 |
| 2APB | 110 | 57 | 15 | 5.04 |
| 1F6L | 114 | 57 | 15 | 5.04 |
| 1ECY | 142 | 57 | 15 | 5.04 |
| 1EWX | 145 | 114 | 30 | 5.04 |
| 1QLX | 210 | 95 | 25 | 5.04 |
| 2AXO | 270 | 152 | 40 | 5.04 |
| 1B3X | 302 | 266 | 70 | 5.04 |
| 1X8Q | 184 | 118 | 31 | 5.04 |
| 3C76 | 184 | 118 | 31 | 5.04 |
| 1MQH | 263 | 217 | 57 | 5.04 |
| 1PXU | 131 | 99 | 26 | 5.04 |
| 2ANG | 123 | 80 | 21 | 5.05 |
| 1QM3 | 112 | 80 | 21 | 5.05 |
| 1IKJ | 184 | 122 | 32 | 5.05 |
| 2ODT | 328 | 244 | 64 | 5.05 |
| 1CUZ | 214 | 164 | 43 | 5.05 |
| 1CUG | 214 | 164 | 43 | 5.05 |
| 1FFA | 214 | 164 | 43 | 5.05 |
| 1FFB | 214 | 164 | 43 | 5.05 |
| 1ZXQ | 192 | 103 | 27 | 5.05 |
| 1CDU | 178 | 103 | 27 | 5.05 |
| 1YCK | 175 | 126 | 33 | 5.05 |
| 1BRU | 241 | 126 | 33 | 5.05 |
| 1UH8 | 325 | 210 | 55 | 5.05 |
| 1B31 | 302 | 275 | 72 | 5.05 |
| 1ESE | 306 | 233 | 61 | 5.05 |
| 2BQG | 130 | 107 | 28 | 5.05 |
| 1KX8 | 112 | 107 | 28 | 5.05 |
| 3BX6 | 192 | 130 | 34 | 5.05 |
| 2HSO | 129 | 88 | 23 | 5.05 |
| 1HJN | 104 | 88 | 23 | 5.05 |
| 1FC7 | 388 | 310 | 81 | 5.05 |
| 1HBP | 183 | 111 | 29 | 5.05 |
| 2GTF | 180 | 111 | 29 | 5.05 |
| 1L4K | 356 | 333 | 87 | 5.05 |
| 4CMS | 323 | 203 | 53 | 5.05 |
| 1CXW | 60 | 23 | 6 | 5.05 |
| 1K0X | 108 | 46 | 12 | 5.05 |
| 1WK1 | 150 | 69 | 18 | 5.05 |
| 1CUY | 214 | 161 | 42 | 5.05 |
| 1V0M | 313 | 280 | 73 | 5.05 |
| 1SXU | 184 | 119 | 31 | 5.05 |
| 1RJ1 | 151 | 192 | 50 | 5.05 |
| 1E82 | 330 | 192 | 50 | 5.05 |
| 2PQL | 145 | 146 | 38 | 5.05 |
| 2CYK | 129 | 123 | 32 | 5.05 |
| 1HGU | 191 | 123 | 32 | 5.05 |
| 1V0N | 313 | 273 | 71 | 5.05 |
| 1A9V | 129 | 50 | 13 | 5.05 |
| 1BGO | 215 | 150 | 39 | 5.05 |
| 2VIM | 104 | 100 | 26 | 5.05 |
| 1ITO | 256 | 154 | 40 | 5.06 |
| 1TBY | 130 | 104 | 27 | 5.06 |
| 1O7U | 146 | 104 | 27 | 5.06 |
| 2UY4 | 294 | 235 | 61 | 5.06 |
| 1FH8 | 312 | 293 | 76 | 5.06 |
| 1GRT | 478 | 374 | 97 | 5.06 |
| 1JV9 | 58 | 27 | 7 | 5.06 |
| 1JC6 | 65 | 27 | 7 | 5.06 |
| 2TMP | 127 | 54 | 14 | 5.06 |
| 2EU7 | 179 | 108 | 28 | 5.06 |
| 1VER | 111 | 54 | 14 | 5.06 |
| 1HQP | 157 | 108 | 28 | 5.06 |
| 1MQI | 263 | 216 | 56 | 5.06 |
| 3EHS | 476 | 359 | 93 | 5.06 |
| 1XG6 | 187 | 85 | 22 | 5.06 |
| 1OA7 | 214 | 116 | 30 | 5.06 |
| 1ANE | 223 | 116 | 30 | 5.06 |
| 1O7C | 98 | 58 | 15 | 5.06 |
| 1FH7 | 312 | 294 | 76 | 5.06 |
| 1LIT | 144 | 89 | 23 | 5.06 |
| 1ZSL | 238 | 120 | 31 | 5.06 |
| 1X8P | 184 | 120 | 31 | 5.06 |
| 1G2S | 71 | 31 | 8 | 5.06 |
| 1BWM | 249 | 101 | 26 | 5.06 |
| 1CS8 | 316 | 241 | 62 | 5.06 |
| 1VMP | 71 | 35 | 9 | 5.06 |
| 1IJ9 | 196 | 105 | 27 | 5.06 |
| 2NVF | 141 | 70 | 18 | 5.06 |
| 1V0L | 313 | 280 | 72 | 5.06 |
| 2JNT | 108 | 109 | 28 | 5.07 |
| 1ERB | 183 | 109 | 28 | 5.07 |
| 2V13 | 340 | 218 | 56 | 5.07 |
| 1CS6 | 382 | 191 | 49 | 5.07 |
| 2AT3 | 184 | 117 | 30 | 5.07 |
| 2A2P | 129 | 78 | 20 | 5.07 |
| 1B30 | 302 | 273 | 70 | 5.07 |
| 1FC9 | 388 | 312 | 80 | 5.07 |
| 2GRT | 461 | 355 | 91 | 5.07 |
| 1KOI | 184 | 121 | 31 | 5.07 |
| 1U2C | 246 | 125 | 32 | 5.07 |
| 2GV7 | 241 | 129 | 33 | 5.07 |
| 1GH2 | 107 | 90 | 23 | 5.07 |
| 2K56 | 113 | 90 | 23 | 5.07 |
| 2EAT | 994 | 830 | 212 | 5.07 |
| 1TDE | 316 | 231 | 59 | 5.07 |
| 1Z78 | 215 | 141 | 36 | 5.07 |
| 2ERF | 209 | 145 | 37 | 5.07 |
| 5PEP | 326 | 200 | 51 | 5.07 |
| 1SM7 | 109 | 51 | 13 | 5.07 |
| 1L8J | 193 | 161 | 41 | 5.07 |
| 2K13 | 103 | 55 | 14 | 5.07 |
| 1BRP | 182 | 110 | 28 | 5.07 |
| 1WUZ | 103 | 55 | 14 | 5.07 |
| 1CUC | 214 | 165 | 42 | 5.07 |
| 3C77 | 184 | 118 | 30 | 5.08 |
| 2APW | 112 | 59 | 15 | 5.08 |
| 1ZHM | 238 | 122 | 31 | 5.08 |
| 2OQ5 | 232 | 122 | 31 | 5.08 |
| 1DEX | 233 | 185 | 47 | 5.08 |
| 2CKN | 95 | 63 | 16 | 5.08 |
| 1OH1 | 109 | 63 | 16 | 5.08 |
| 2ZD8 | 265 | 260 | 66 | 5.08 |
| 1GIO | 125 | 67 | 17 | 5.08 |
| 1MEK | 120 | 67 | 17 | 5.08 |
| 2ASI | 361 | 201 | 51 | 5.08 |
| 1DIX | 208 | 138 | 35 | 5.08 |
| 2LAO | 238 | 209 | 53 | 5.08 |
| 2NUK | 141 | 71 | 18 | 5.08 |
| 1UKZ | 203 | 213 | 54 | 5.08 |
| 1AU0 | 215 | 150 | 38 | 5.08 |
| 1QDQ | 253 | 154 | 39 | 5.08 |
| 1AYU | 215 | 154 | 39 | 5.08 |
| 1CUX | 214 | 162 | 41 | 5.08 |
| 1XKU | 330 | 166 | 42 | 5.08 |
| 1UFU | 197 | 87 | 22 | 5.08 |
| 2HIS | 312 | 265 | 67 | 5.08 |
| 1ZJK | 403 | 190 | 48 | 5.08 |
| 1FQ6 | 329 | 198 | 50 | 5.08 |
| 2VS2 | 329 | 198 | 50 | 5.08 |
| 1V0S | 506 | 408 | 103 | 5.08 |
| 2O6W | 150 | 103 | 26 | 5.08 |
| 1TCY | 130 | 107 | 27 | 5.08 |
| 1QQY | 130 | 107 | 27 | 5.08 |
| 1DF3 | 162 | 107 | 27 | 5.08 |
| 2OVE | 182 | 107 | 27 | 5.08 |
| 3PSG | 370 | 234 | 59 | 5.08 |
| 1D2U | 184 | 119 | 30 | 5.08 |
| 1ML7 | 184 | 119 | 30 | 5.08 |
| 1D3S | 184 | 119 | 30 | 5.08 |
| 2Z8T | 185 | 135 | 34 | 5.08 |
| 1FH9 | 312 | 290 | 73 | 5.08 |
| 2P86 | 215 | 147 | 37 | 5.08 |
| 1Z15 | 344 | 306 | 77 | 5.08 |
| 1CUU | 214 | 163 | 41 | 5.09 |
| 1FFC | 214 | 163 | 41 | 5.09 |
| 1XCD | 329 | 163 | 41 | 5.09 |
| 1SLY | 618 | 656 | 165 | 5.09 |
| 1US2 | 530 | 382 | 96 | 5.09 |
| 4APE | 330 | 195 | 49 | 5.09 |
| 2EAR | 994 | 745 | 187 | 5.09 |
| 1B3Z | 302 | 267 | 67 | 5.09 |
| 1RO3 | 49 | 12 | 3 | 5.09 |
| 1BF0 | 60 | 20 | 5 | 5.09 |
| 1PDC | 45 | 16 | 4 | 5.09 |
| 1B2I | 83 | 24 | 6 | 5.09 |
| 1HHV | 74 | 32 | 8 | 5.09 |
| 1U6Q | 245 | 132 | 33 | 5.09 |
| 1GA3 | 113 | 80 | 20 | 5.09 |
| 1XJT | 191 | 148 | 37 | 5.09 |
| 2GW5 | 197 | 92 | 23 | 5.09 |
| 1UGN | 198 | 92 | 23 | 5.09 |
| 1R4Y | 136 | 48 | 12 | 5.09 |
| 1AU2 | 215 | 136 | 34 | 5.09 |
| 1EPR | 330 | 192 | 48 | 5.09 |
| 1P5Y | 548 | 212 | 53 | 5.09 |
| 3B43 | 570 | 301 | 75 | 5.09 |
| 2UY2 | 294 | 237 | 59 | 5.09 |
| 2UY3 | 294 | 229 | 57 | 5.09 |
| 1LBB | 263 | 221 | 55 | 5.10 |
| 1YX9 | 326 | 209 | 52 | 5.10 |
| 3CMS | 323 | 205 | 51 | 5.10 |
| 2P7U | 215 | 145 | 36 | 5.10 |
| 1BQI | 212 | 137 | 34 | 5.10 |
| 2UY5 | 294 | 230 | 57 | 5.10 |
| 1BCN | 133 | 109 | 27 | 5.10 |
| 1YTK | 398 | 315 | 78 | 5.10 |
| 1XEZ | 721 | 404 | 100 | 5.10 |
| 1FQ4 | 329 | 194 | 48 | 5.10 |
| 1J01 | 312 | 287 | 71 | 5.10 |
| 2EA1 | 245 | 186 | 46 | 5.10 |
| 1B3W | 302 | 275 | 68 | 5.10 |
| 1L35 | 164 | 182 | 45 | 5.10 |
| 1B3Y | 302 | 271 | 67 | 5.10 |
| 3ETP | 187 | 89 | 22 | 5.10 |
| 1E1P | 104 | 89 | 22 | 5.10 |
| 1R8N | 185 | 85 | 21 | 5.10 |
| 1DBY | 107 | 81 | 20 | 5.10 |
| 1Q25 | 432 | 231 | 57 | 5.10 |
| 1XMW | 178 | 77 | 19 | 5.10 |
| 2D80 | 318 | 227 | 56 | 5.10 |
| 1VCZ | 217 | 150 | 37 | 5.10 |
| 2NUM | 141 | 69 | 17 | 5.10 |
| 1AC5 | 483 | 370 | 91 | 5.11 |
| 2RG3 | 218 | 114 | 28 | 5.11 |
| 1KT7 | 183 | 114 | 28 | 5.11 |
| 1DE1 | 87 | 57 | 14 | 5.11 |
| 2APX | 112 | 57 | 14 | 5.11 |
| 2APF | 112 | 57 | 14 | 5.11 |
| 1T68 | 180 | 110 | 27 | 5.11 |
| 1EZK | 153 | 110 | 27 | 5.11 |
| 1IW2 | 182 | 110 | 27 | 5.11 |
| 1NKR | 201 | 106 | 26 | 5.11 |
| 2JCP | 154 | 102 | 25 | 5.11 |
| 1QK8 | 146 | 102 | 25 | 5.11 |
| 2P52 | 196 | 102 | 25 | 5.11 |
| 1TDG | 265 | 253 | 62 | 5.11 |
| 2CE6 | 101 | 49 | 12 | 5.11 |
| 2B5Y | 148 | 98 | 24 | 5.11 |
| 1US4 | 314 | 241 | 59 | 5.11 |
| 1B8E | 162 | 94 | 23 | 5.11 |
| 1CNV | 299 | 233 | 57 | 5.11 |
| 1I1X | 303 | 274 | 67 | 5.11 |
| 1JNJ | 100 | 45 | 11 | 5.11 |
| 2BSB | 176 | 90 | 22 | 5.11 |
| 2ANJ | 263 | 221 | 54 | 5.11 |
| 2JKQ | 276 | 213 | 52 | 5.11 |
| 2H0T | 265 | 254 | 62 | 5.11 |
| 2H0Y | 265 | 254 | 62 | 5.11 |
| 1UKY | 203 | 209 | 51 | 5.11 |
| 2OFS | 75 | 41 | 10 | 5.11 |
| 1THX | 115 | 82 | 20 | 5.11 |
| 1WWC | 118 | 41 | 10 | 5.11 |
| 1LAF | 238 | 205 | 50 | 5.11 |
| 2HKY | 129 | 78 | 19 | 5.11 |
| 1W8K | 447 | 222 | 54 | 5.12 |
| 2REN | 340 | 185 | 45 | 5.12 |
| 1B6U | 257 | 111 | 27 | 5.12 |
| 1KFW | 435 | 333 | 81 | 5.12 |
| 1YK8 | 215 | 144 | 35 | 5.12 |
| 1F42 | 306 | 144 | 35 | 5.12 |
| 2HLV | 160 | 107 | 26 | 5.12 |
| 1ULO | 152 | 70 | 17 | 5.12 |
| 1V0Y | 506 | 408 | 99 | 5.12 |
| 2UYT | 489 | 441 | 107 | 5.12 |
| 1EMR | 159 | 169 | 41 | 5.12 |
| 1LAG | 238 | 202 | 49 | 5.12 |
| 1FHD | 312 | 297 | 72 | 5.12 |
| 1QG5 | 162 | 99 | 24 | 5.12 |
| 1CDI | 179 | 95 | 23 | 5.12 |
| 1KXO | 184 | 95 | 23 | 5.12 |
| 1AYV | 215 | 157 | 38 | 5.12 |
| 2OK5 | 752 | 434 | 105 | 5.12 |
| 1JPE | 151 | 62 | 15 | 5.12 |
| 1AVU | 181 | 91 | 22 | 5.12 |
| 1DR9 | 201 | 120 | 29 | 5.12 |
| 1GUV | 366 | 294 | 71 | 5.12 |
| 1LAH | 238 | 203 | 49 | 5.12 |
| 1HA0 | 494 | 340 | 82 | 5.12 |
| 1M5C | 263 | 220 | 53 | 5.12 |
| 1SGL | 209 | 137 | 33 | 5.12 |
| 1OWI | 245 | 137 | 33 | 5.12 |
| 1SFP | 114 | 54 | 13 | 5.12 |
| 1F0N | 285 | 237 | 57 | 5.13 |
| 2E4Z | 501 | 329 | 79 | 5.13 |
| 2EAS | 994 | 754 | 181 | 5.13 |
| 1ZFL | 67 | 25 | 6 | 5.13 |
| 1AWZ | 123 | 75 | 18 | 5.13 |
| 1K58 | 123 | 75 | 18 | 5.13 |
| 1ATK | 215 | 150 | 36 | 5.13 |
| 1YK7 | 215 | 150 | 36 | 5.13 |
| 1ARC | 268 | 146 | 35 | 5.13 |
| 1ITL | 130 | 121 | 29 | 5.13 |
| 2AE1 | 260 | 238 | 57 | 5.13 |
| 1TOL | 222 | 117 | 28 | 5.13 |
| 1UCA | 190 | 138 | 33 | 5.13 |
| 1O9W | 177 | 92 | 22 | 5.13 |
| 2ZJM | 405 | 226 | 54 | 5.13 |
| 1IKO | 178 | 67 | 16 | 5.13 |
| 1A7M | 180 | 155 | 37 | 5.13 |
| 1O9V | 177 | 88 | 21 | 5.13 |
| 1BJ7 | 156 | 109 | 26 | 5.13 |
| 2RKC | 462 | 218 | 52 | 5.13 |
| 2OVD | 182 | 109 | 26 | 5.13 |
| 1J7M | 72 | 21 | 5 | 5.13 |
| 1MEM | 215 | 147 | 35 | 5.13 |
| 1F2C | 215 | 126 | 30 | 5.13 |
| 2H10 | 265 | 252 | 60 | 5.13 |
| 1ONG | 265 | 252 | 60 | 5.13 |
| 1ONF | 500 | 328 | 78 | 5.14 |
| 1LN1 | 214 | 143 | 34 | 5.14 |
| 2OUJ | 251 | 143 | 34 | 5.14 |
| 2B8L | 405 | 223 | 53 | 5.14 |
| 1TIE | 172 | 80 | 19 | 5.14 |
| 1UCC | 190 | 139 | 33 | 5.14 |
| 1MS6 | 222 | 156 | 37 | 5.14 |
| 2DVK | 188 | 156 | 37 | 5.14 |
| 1I6A | 219 | 156 | 37 | 5.14 |
| 1G0X | 197 | 97 | 23 | 5.14 |
| 1SQT | 245 | 135 | 32 | 5.14 |
| 1UAI | 224 | 135 | 32 | 5.14 |
| 1TDL | 265 | 249 | 59 | 5.14 |
| 2E59 | 144 | 76 | 18 | 5.14 |
| 1CTO | 109 | 38 | 9 | 5.14 |
| 1FM5 | 199 | 76 | 18 | 5.14 |
| 1I04 | 180 | 114 | 27 | 5.14 |
| 2ZF8 | 278 | 152 | 36 | 5.14 |
| 1XYW | 111 | 93 | 22 | 5.14 |
| 1CEG | 349 | 292 | 69 | 5.14 |
| 2A3U | 270 | 254 | 60 | 5.14 |
| 1CIV | 385 | 305 | 72 | 5.14 |
| 1IY3 | 130 | 89 | 21 | 5.14 |
| 1DWZ | 112 | 89 | 21 | 5.14 |
| 2BSC | 177 | 89 | 21 | 5.14 |
| 1ME3 | 215 | 123 | 29 | 5.14 |
| 1VM1 | 265 | 246 | 58 | 5.14 |
| 1AYW | 215 | 140 | 33 | 5.14 |
| 2LIV | 344 | 280 | 66 | 5.14 |
| 3PTE | 349 | 297 | 70 | 5.14 |
| 1N1F | 159 | 174 | 41 | 5.14 |
| 1NJR | 284 | 174 | 41 | 5.14 |
| 1FQ8 | 329 | 191 | 45 | 5.14 |
| 1AIM | 215 | 136 | 32 | 5.14 |
| 1NN6 | 228 | 119 | 28 | 5.14 |
| 2G0F | 168 | 119 | 28 | 5.14 |
| 1CRL | 534 | 408 | 96 | 5.14 |
| 1YTD | 398 | 319 | 75 | 5.15 |
| 1Z17 | 344 | 319 | 75 | 5.15 |
| 1YSC | 421 | 302 | 71 | 5.15 |
| 2MAN | 302 | 251 | 59 | 5.15 |
| 1IDK | 359 | 234 | 55 | 5.15 |
| 2BAA | 243 | 217 | 51 | 5.15 |
| 2A5S | 284 | 217 | 51 | 5.15 |
| 1Y15 | 112 | 98 | 23 | 5.15 |
| 1FQ5 | 329 | 196 | 46 | 5.15 |
| 1GNV | 266 | 196 | 46 | 5.15 |
| 1SKB | 439 | 375 | 88 | 5.15 |
| 1QM2 | 112 | 81 | 19 | 5.15 |
| 1PW1 | 349 | 290 | 68 | 5.15 |
| 2HAI | 576 | 499 | 117 | 5.15 |
| 2EX6 | 458 | 354 | 83 | 5.15 |
| 1S6N | 115 | 64 | 15 | 5.15 |
| 1OD7 | 119 | 64 | 15 | 5.15 |
| 1CEF | 349 | 286 | 67 | 5.15 |
| 1UWF | 158 | 94 | 22 | 5.15 |
| 1O8M | 353 | 231 | 54 | 5.15 |
| 3BQI | 360 | 291 | 68 | 5.15 |
| 2OVA | 182 | 107 | 25 | 5.15 |
| 1JWF | 147 | 167 | 39 | 5.15 |
| 1KIV | 78 | 30 | 7 | 5.15 |
| 1RJT | 73 | 30 | 7 | 5.15 |
| 1O9Z | 177 | 90 | 21 | 5.15 |
| 1DX1 | 219 | 90 | 21 | 5.15 |
| 3BUL | 579 | 523 | 122 | 5.15 |
| 2D81 | 318 | 223 | 52 | 5.15 |
| 1RCB | 129 | 133 | 31 | 5.15 |
| 1H4U | 265 | 159 | 37 | 5.15 |
| 3C5A | 264 | 245 | 57 | 5.15 |
| 1EWL | 215 | 129 | 30 | 5.15 |
| 1CPY | 421 | 301 | 70 | 5.15 |
| 2HVM | 273 | 215 | 50 | 5.15 |
| 1Z24 | 189 | 129 | 30 | 5.15 |
| 1M4B | 133 | 129 | 30 | 5.15 |
| 1WNS | 774 | 529 | 123 | 5.15 |
| 2F7D | 215 | 142 | 33 | 5.15 |
| 1XYU | 111 | 99 | 23 | 5.16 |
| 1KQZ | 273 | 211 | 49 | 5.16 |
| 1ME4 | 215 | 125 | 29 | 5.16 |
| 1MEG | 216 | 138 | 32 | 5.16 |
| 1BY8 | 314 | 207 | 48 | 5.16 |
| 1QUW | 105 | 69 | 16 | 5.16 |
| 1RIE | 129 | 69 | 16 | 5.16 |
| 2B8V | 405 | 220 | 51 | 5.16 |
| 3FKT | 405 | 220 | 51 | 5.16 |
| 1Q2P | 265 | 246 | 57 | 5.16 |
| 1PWG | 349 | 298 | 69 | 5.16 |
| 3C9X | 329 | 203 | 47 | 5.16 |
| 2EX9 | 458 | 350 | 81 | 5.16 |
| 1ZHR | 238 | 121 | 28 | 5.16 |
| 1ARB | 268 | 147 | 34 | 5.16 |
| 1CMS | 323 | 212 | 49 | 5.16 |
| 1TQF | 405 | 225 | 52 | 5.16 |
| 1EL0 | 74 | 26 | 6 | 5.16 |
| 2RLP | 129 | 52 | 12 | 5.16 |
| 1CFI | 47 | 13 | 3 | 5.16 |
| 1W12 | 247 | 117 | 27 | 5.16 |
| 1POZ | 159 | 91 | 21 | 5.16 |
| 2AIM | 215 | 130 | 30 | 5.16 |
| 3C78 | 184 | 117 | 27 | 5.16 |
| 3NCM | 92 | 52 | 12 | 5.16 |
| 3CAF | 100 | 52 | 12 | 5.16 |
| 1LQ0 | 365 | 299 | 69 | 5.16 |
| 1E81 | 330 | 195 | 45 | 5.16 |
| 1Y9U | 323 | 282 | 65 | 5.16 |
| 1LLO | 273 | 217 | 50 | 5.16 |
| 2EX8 | 458 | 369 | 85 | 5.16 |
| 1N9B | 265 | 252 | 58 | 5.16 |
| 3MAN | 302 | 252 | 58 | 5.16 |
| 1TCA | 317 | 239 | 55 | 5.16 |
| 1RD6 | 563 | 400 | 92 | 5.16 |
| 1MP8 | 281 | 222 | 51 | 5.16 |
| 1EI9 | 279 | 209 | 48 | 5.16 |
| 1WRF | 129 | 61 | 14 | 5.17 |
| 1ZOX | 113 | 61 | 14 | 5.17 |
| 1SI5 | 240 | 122 | 28 | 5.17 |
| 2NMS | 124 | 61 | 14 | 5.17 |
| 2ZWJ | 152 | 183 | 42 | 5.17 |
| 2HHI | 204 | 122 | 28 | 5.17 |
| 1SHV | 265 | 244 | 56 | 5.17 |
| 1HVQ | 273 | 205 | 47 | 5.17 |
| 1GH5 | 87 | 48 | 11 | 5.17 |
| 1AX8 | 146 | 144 | 33 | 5.17 |
| 1K6A | 303 | 275 | 63 | 5.17 |
| 2NTR | 405 | 227 | 52 | 5.17 |
| 1KS9 | 291 | 262 | 60 | 5.17 |
| 1ZHP | 238 | 118 | 27 | 5.17 |
| 1GOK | 303 | 271 | 62 | 5.17 |
| 2A49 | 269 | 258 | 59 | 5.17 |
| 2DIZ | 117 | 70 | 16 | 5.17 |
| 3BUG | 409 | 210 | 48 | 5.17 |
| 3B9E | 584 | 420 | 96 | 5.17 |
| 2PLF | 414 | 245 | 56 | 5.17 |
| 1QCX | 359 | 241 | 55 | 5.17 |
| 3DCN | 201 | 149 | 34 | 5.17 |
| 1NH6 | 540 | 399 | 91 | 5.17 |
| 1EH5 | 279 | 215 | 49 | 5.17 |
| 2P8H | 405 | 224 | 51 | 5.17 |
| 1EWM | 215 | 123 | 28 | 5.17 |
| 3B9A | 584 | 413 | 94 | 5.17 |
| 1YAL | 218 | 145 | 33 | 5.17 |
| 3CD1 | 248 | 145 | 33 | 5.17 |
| 2HTQ | 390 | 189 | 43 | 5.17 |
| 1K9T | 540 | 400 | 91 | 5.17 |
| 1F0P | 285 | 233 | 53 | 5.17 |
| 1EWO | 215 | 132 | 30 | 5.17 |
| 2EXA | 458 | 352 | 80 | 5.17 |
| 2AIX | 263 | 220 | 50 | 5.17 |
| 1RCJ | 265 | 251 | 57 | 5.17 |
| 2B94 | 267 | 207 | 47 | 5.17 |
| 2JKO | 276 | 216 | 49 | 5.18 |
| 1GL8 | 104 | 75 | 17 | 5.18 |
| 1SZN | 417 | 300 | 68 | 5.18 |
| 2G5D | 422 | 256 | 58 | 5.18 |
| 1AOV | 686 | 490 | 111 | 5.18 |
| 1FQ7 | 329 | 181 | 41 | 5.18 |
| 2CGJ | 489 | 415 | 94 | 5.18 |
| 1NQC | 217 | 159 | 36 | 5.18 |
| 2BLG | 162 | 106 | 24 | 5.18 |
| 2QZL | 411 | 221 | 50 | 5.18 |
| 2SIM | 381 | 221 | 50 | 5.18 |
| 1EIB | 540 | 389 | 88 | 5.18 |
| 1OKD | 154 | 84 | 19 | 5.18 |
| 2H5S | 265 | 252 | 57 | 5.18 |
| 1TRH | 534 | 398 | 90 | 5.18 |
| 1O8I | 353 | 230 | 52 | 5.18 |
| 1XQP | 256 | 261 | 59 | 5.18 |
| 1MD8 | 329 | 177 | 40 | 5.18 |
| 1D2B | 126 | 62 | 14 | 5.18 |
| 1DV9 | 162 | 93 | 21 | 5.18 |
| 1NEU | 124 | 62 | 14 | 5.18 |
| 1X3K | 152 | 186 | 42 | 5.18 |
| 2IXG | 271 | 186 | 42 | 5.18 |
| 1CF3 | 583 | 461 | 104 | 5.18 |
| 1IEX | 605 | 470 | 106 | 5.18 |
| 1HIK | 129 | 142 | 32 | 5.18 |
| 2NWF | 141 | 71 | 16 | 5.18 |
| 1GAH | 471 | 435 | 98 | 5.18 |
| 1PE6 | 212 | 151 | 34 | 5.18 |
| 2PEC | 353 | 231 | 52 | 5.18 |
| 1KLT | 226 | 120 | 27 | 5.18 |
| 1MPL | 349 | 298 | 67 | 5.18 |
| 2BSX | 253 | 178 | 40 | 5.18 |
| 1PPN | 212 | 147 | 33 | 5.18 |
| 1IWD | 215 | 147 | 33 | 5.18 |
| 2A4H | 126 | 49 | 11 | 5.18 |
| 2JJW | 127 | 49 | 11 | 5.18 |
| 1KS5 | 223 | 147 | 33 | 5.18 |
| 2EXB | 458 | 352 | 79 | 5.18 |
| 1GLO | 217 | 156 | 35 | 5.18 |
| 2RIK | 284 | 156 | 35 | 5.18 |
| 2JS7 | 160 | 107 | 24 | 5.18 |
| 1LG1 | 365 | 281 | 63 | 5.18 |
| 3BZ3 | 276 | 232 | 52 | 5.18 |
| 1BP4 | 212 | 143 | 32 | 5.19 |
| 1TM2 | 316 | 286 | 64 | 5.19 |
| 2QZK | 405 | 228 | 51 | 5.19 |
| 3B9D | 584 | 416 | 93 | 5.19 |
| 1JLI | 112 | 85 | 19 | 5.19 |
| 2D3J | 157 | 85 | 19 | 5.19 |
| 2DLI | 197 | 94 | 21 | 5.19 |
| 1TJY | 316 | 282 | 63 | 5.19 |
| 4PAD | 212 | 121 | 27 | 5.19 |
| 1SDE | 347 | 296 | 66 | 5.19 |
| 1IKG | 349 | 296 | 66 | 5.19 |
| 1APA | 266 | 211 | 47 | 5.19 |
| 1F2Q | 176 | 81 | 18 | 5.19 |
| 2NVE | 141 | 72 | 16 | 5.19 |
| 2Z63 | 570 | 360 | 80 | 5.19 |
| 1QM1 | 143 | 90 | 20 | 5.19 |
| 2JMR | 179 | 63 | 14 | 5.19 |
| 3EMY | 329 | 198 | 44 | 5.19 |
| 1GOO | 303 | 275 | 61 | 5.19 |
| 2C0Y | 315 | 257 | 57 | 5.19 |
| 1GAI | 472 | 442 | 98 | 5.19 |
| 2JKM | 276 | 212 | 47 | 5.19 |
| 1V9M | 323 | 361 | 80 | 5.19 |
| 1F2A | 215 | 131 | 29 | 5.20 |
| 1JB9 | 316 | 226 | 50 | 5.20 |
| 2R5G | 247 | 208 | 46 | 5.20 |
| 1B10 | 142 | 95 | 21 | 5.20 |
| 2PER | 267 | 190 | 42 | 5.20 |
| 3F5F | 658 | 543 | 120 | 5.20 |
| 1CJL | 312 | 240 | 53 | 5.20 |
| 1HQ8 | 123 | 77 | 17 | 5.20 |
| 2ZB6 | 481 | 231 | 51 | 5.20 |
| 2D43 | 482 | 308 | 68 | 5.20 |
| 1RXL | 156 | 77 | 17 | 5.20 |
| 2D44 | 482 | 299 | 66 | 5.20 |
| 1V6Y | 324 | 299 | 66 | 5.20 |
| 1N1Y | 641 | 367 | 81 | 5.20 |
| 1HKI | 365 | 290 | 64 | 5.20 |
| 1BK7 | 190 | 136 | 30 | 5.20 |
| 2NLR | 234 | 136 | 30 | 5.20 |
| 2VQC | 118 | 68 | 15 | 5.20 |
| 1ULP | 152 | 68 | 15 | 5.20 |
| 2BNJ | 303 | 272 | 60 | 5.20 |
| 1IFG | 140 | 59 | 13 | 5.20 |
| 2J7L | 218 | 109 | 24 | 5.20 |
| 2IL6 | 185 | 159 | 35 | 5.20 |
| 2VJ7 | 392 | 223 | 49 | 5.20 |
| 1W81 | 447 | 214 | 47 | 5.20 |
| 2ZJJ | 405 | 214 | 47 | 5.20 |
| 2VN4 | 599 | 510 | 112 | 5.20 |
| 1PW8 | 349 | 296 | 65 | 5.20 |
| 1YQS | 349 | 296 | 65 | 5.20 |
| 1HVB | 349 | 296 | 65 | 5.20 |
| 2QV3 | 457 | 296 | 65 | 5.20 |
| 1AGI | 125 | 82 | 18 | 5.20 |
| 1DOY | 96 | 41 | 9 | 5.20 |
| 1ALY | 146 | 82 | 18 | 5.20 |
| 1HIJ | 129 | 137 | 30 | 5.20 |
| 1M1H | 248 | 137 | 30 | 5.20 |
| 1SCW | 349 | 297 | 65 | 5.20 |
| 1P6S | 111 | 64 | 14 | 5.20 |
| 2VUJ | 219 | 128 | 28 | 5.20 |
| 1PZO | 263 | 247 | 54 | 5.21 |
| 3E8W | 220 | 183 | 40 | 5.21 |
| 1FFQ | 540 | 389 | 85 | 5.21 |
| 2B1K | 168 | 119 | 26 | 5.21 |
| 1HKJ | 365 | 293 | 64 | 5.21 |
| 1PWC | 349 | 293 | 64 | 5.21 |
| 1PWD | 349 | 293 | 64 | 5.21 |
| 1MD7 | 328 | 142 | 31 | 5.21 |
| 3PBH | 317 | 197 | 43 | 5.21 |
| 1MWP | 96 | 55 | 12 | 5.21 |
| 1H3W | 223 | 110 | 24 | 5.21 |
| 1I9E | 115 | 55 | 12 | 5.21 |
| 2VJ9 | 392 | 220 | 48 | 5.21 |
| 2NYK | 285 | 188 | 41 | 5.21 |
| 1IQQ | 200 | 156 | 34 | 5.21 |
| 1BUE | 265 | 234 | 51 | 5.21 |
| 1EDQ | 540 | 390 | 85 | 5.21 |
| 2VIY | 392 | 225 | 49 | 5.21 |
| 2W62 | 555 | 317 | 69 | 5.21 |
| 1CTN | 540 | 386 | 84 | 5.21 |
| 2HTR | 390 | 207 | 45 | 5.21 |
| 2PBH | 317 | 184 | 40 | 5.21 |
| 2FYE | 217 | 138 | 30 | 5.21 |
| 1PJW | 111 | 46 | 10 | 5.21 |
| 1Y16 | 112 | 92 | 20 | 5.21 |
| 1XS7 | 389 | 230 | 50 | 5.21 |
| 2BS8 | 176 | 92 | 20 | 5.21 |
| 3E8T | 220 | 184 | 40 | 5.21 |
| 1SGK | 535 | 414 | 90 | 5.21 |
| 2IRZ | 405 | 221 | 48 | 5.21 |
| 1I39 | 225 | 198 | 43 | 5.21 |
| 1EHN | 540 | 387 | 84 | 5.21 |
| 1HKM | 365 | 295 | 64 | 5.21 |
| 1PPP | 212 | 143 | 31 | 5.21 |
| 1ITX | 419 | 346 | 75 | 5.21 |
| 1ITM | 130 | 120 | 26 | 5.21 |
| 1X6N | 563 | 402 | 87 | 5.21 |
| 1J1R | 261 | 208 | 45 | 5.21 |
| 3EGP | 108 | 37 | 8 | 5.21 |
| 1VKB | 161 | 88 | 19 | 5.21 |
| 2EAU | 994 | 857 | 185 | 5.22 |
| 1CK3 | 263 | 241 | 52 | 5.22 |
| 1TDK | 486 | 399 | 86 | 5.22 |
| 1GLM | 470 | 427 | 92 | 5.22 |
| 1MRY | 339 | 195 | 42 | 5.22 |
| 9PAP | 212 | 144 | 31 | 5.22 |
| 2OAY | 390 | 288 | 62 | 5.22 |
| 2EX2 | 458 | 367 | 79 | 5.22 |
| 2YZU | 109 | 79 | 17 | 5.22 |
| 3GLY | 470 | 423 | 91 | 5.22 |
| 1HUW | 191 | 172 | 37 | 5.22 |
| 1QQS | 174 | 107 | 23 | 5.22 |
| 2OAH | 405 | 228 | 49 | 5.22 |
| 2PAD | 212 | 121 | 26 | 5.22 |
| 1X6L | 563 | 396 | 85 | 5.22 |
| 2ANW | 241 | 126 | 27 | 5.22 |
| 3C8X | 206 | 84 | 18 | 5.22 |
| 2VJ6 | 392 | 224 | 48 | 5.22 |
| 4TSV | 150 | 70 | 15 | 5.22 |
| 1PZP | 263 | 252 | 54 | 5.22 |
| 1TDN | 486 | 402 | 86 | 5.22 |
| 2I6S | 517 | 346 | 74 | 5.22 |
| 2ZIY | 372 | 318 | 68 | 5.22 |
| 1FMU | 329 | 206 | 44 | 5.22 |
| 1OGM | 574 | 309 | 66 | 5.22 |
| 2B1M | 246 | 150 | 32 | 5.22 |
| 1LG2 | 365 | 300 | 64 | 5.22 |
| 1AVK | 638 | 361 | 77 | 5.22 |
| 2HVX | 226 | 122 | 26 | 5.23 |
| 1SKA | 439 | 366 | 78 | 5.23 |
| 1DOG | 470 | 427 | 91 | 5.23 |
| 1S50 | 259 | 216 | 46 | 5.23 |
| 1AEC | 218 | 155 | 33 | 5.23 |
| 2Z5J | 890 | 794 | 169 | 5.23 |
| 3D4S | 490 | 456 | 97 | 5.23 |
| 1WD4 | 482 | 301 | 64 | 5.23 |
| 1F2B | 215 | 127 | 27 | 5.23 |
| 2JF4 | 535 | 381 | 81 | 5.23 |
| 1BQC | 302 | 259 | 55 | 5.23 |
| 2SDF | 67 | 33 | 7 | 5.23 |
| 2FHT | 71 | 33 | 7 | 5.23 |
| 2AEY | 543 | 297 | 63 | 5.23 |
| 2DL2 | 197 | 85 | 18 | 5.23 |
| 1FFR | 540 | 392 | 83 | 5.23 |
| 1OM0 | 274 | 222 | 47 | 5.23 |
| 1AGM | 470 | 430 | 91 | 5.23 |
| 2VGA | 207 | 104 | 22 | 5.23 |
| 2ZJN | 405 | 208 | 44 | 5.23 |
| 1GQZ | 274 | 194 | 41 | 5.23 |
| 1VD3 | 217 | 142 | 30 | 5.23 |
| 1NYL | 539 | 341 | 72 | 5.23 |
| 1ZK5 | 176 | 90 | 19 | 5.23 |
| 1GOQ | 303 | 270 | 57 | 5.23 |
| 1BY3 | 714 | 469 | 99 | 5.23 |
| 2W63 | 555 | 313 | 66 | 5.23 |
| 1GOM | 303 | 266 | 56 | 5.23 |
| 1YON | 303 | 271 | 57 | 5.24 |
| 2ZHS | 411 | 214 | 45 | 5.24 |
| 2HT7 | 390 | 195 | 41 | 5.24 |
| 2IY9 | 347 | 238 | 50 | 5.24 |
| 2ZJL | 405 | 200 | 42 | 5.24 |
| 2CZK | 299 | 200 | 42 | 5.24 |
| 1RN7 | 122 | 81 | 17 | 5.24 |
| 1VD1 | 217 | 143 | 30 | 5.24 |
| 2Q15 | 385 | 205 | 43 | 5.24 |
| 1FCU | 350 | 267 | 56 | 5.24 |
| 1KUM | 108 | 62 | 13 | 5.24 |
| 1THG | 544 | 434 | 91 | 5.24 |
| 2QET | 261 | 229 | 48 | 5.24 |
| 1GOR | 303 | 272 | 57 | 5.24 |
| 2IM9 | 333 | 253 | 53 | 5.24 |
| 1PPD | 212 | 148 | 31 | 5.24 |
| 1IKI | 349 | 296 | 62 | 5.24 |
| 2FCB | 173 | 86 | 18 | 5.24 |
| 1E4J | 176 | 86 | 18 | 5.24 |
| 1J1S | 261 | 215 | 45 | 5.24 |
| 1MZ5 | 638 | 387 | 81 | 5.24 |
| 2OYE | 600 | 397 | 83 | 5.24 |
| 1REO | 486 | 402 | 84 | 5.24 |
| 1BWZ | 274 | 182 | 38 | 5.24 |
| 1ZTY | 529 | 388 | 81 | 5.24 |
| 2ZE1 | 415 | 211 | 44 | 5.24 |
| 1EX1 | 605 | 470 | 98 | 5.24 |
| 2HT8 | 390 | 192 | 40 | 5.24 |
| 2JSF | 117 | 48 | 10 | 5.24 |
| 2A6Z | 222 | 120 | 25 | 5.24 |
| 2ECF | 741 | 442 | 92 | 5.24 |
| 1WD3 | 482 | 298 | 62 | 5.24 |
| 2Z53 | 261 | 226 | 47 | 5.24 |
| 2Z82 | 549 | 327 | 68 | 5.24 |
| 1T0O | 417 | 308 | 64 | 5.24 |
| 2Z4U | 261 | 231 | 48 | 5.24 |
| 2QES | 261 | 231 | 48 | 5.24 |
| 1NLR | 234 | 130 | 27 | 5.24 |
| 1X42 | 232 | 212 | 44 | 5.25 |
| 1YR8 | 262 | 212 | 44 | 5.25 |
| 3BRA | 409 | 217 | 45 | 5.25 |
| 1J1G | 190 | 140 | 29 | 5.25 |
| 2HTU | 390 | 198 | 41 | 5.25 |
| 2IQG | 406 | 227 | 47 | 5.25 |
| 3DJ9 | 107 | 58 | 12 | 5.25 |
| 1EAG | 342 | 203 | 42 | 5.25 |
| 1EXW | 279 | 218 | 45 | 5.25 |
| 1QSZ | 101 | 63 | 13 | 5.25 |
| 1UZ2 | 162 | 97 | 20 | 5.25 |
| 1WDY | 285 | 291 | 60 | 5.25 |
| 1XOE | 387 | 199 | 41 | 5.25 |
| 3E0G | 483 | 199 | 41 | 5.25 |
| 2Z4F | 173 | 68 | 14 | 5.25 |
| 1OA4 | 222 | 136 | 28 | 5.25 |
| 1J1Q | 261 | 209 | 43 | 5.25 |
| 2ZJI | 405 | 209 | 43 | 5.25 |
| 153L | 185 | 185 | 38 | 5.25 |
| 2VNN | 392 | 224 | 46 | 5.25 |
| 2VNM | 392 | 229 | 47 | 5.25 |
| 1MIT | 69 | 39 | 8 | 5.25 |
| 2PB1 | 400 | 317 | 65 | 5.25 |
| 1U9Q | 215 | 127 | 26 | 5.26 |
| 1VHU | 211 | 171 | 35 | 5.26 |
| 2DE0 | 526 | 391 | 80 | 5.26 |
| 2VSD | 105 | 44 | 9 | 5.26 |
| 2IS0 | 405 | 225 | 46 | 5.26 |
| 1EQP | 394 | 318 | 65 | 5.26 |
| 2HTW | 390 | 186 | 38 | 5.26 |
| 1E1S | 104 | 93 | 19 | 5.26 |
| 1BUL | 265 | 235 | 48 | 5.26 |
| 2OLH | 361 | 284 | 58 | 5.26 |
| 2DSU | 361 | 289 | 59 | 5.26 |
| 2CGZ | 101 | 49 | 10 | 5.26 |
| 1DDT | 535 | 392 | 80 | 5.26 |
| 1BY7 | 382 | 294 | 60 | 5.26 |
| 1AXB | 263 | 250 | 51 | 5.26 |
| 1NYY | 263 | 250 | 51 | 5.26 |
| 1CBG | 490 | 402 | 82 | 5.26 |
| 1O3U | 135 | 157 | 32 | 5.26 |
| 1N5P | 105 | 54 | 11 | 5.26 |
| 1KUL | 108 | 54 | 11 | 5.26 |
| 1H12 | 405 | 388 | 79 | 5.26 |
| 1RH9 | 373 | 344 | 70 | 5.26 |
| 1GAL | 583 | 462 | 94 | 5.26 |
| 1UN2 | 197 | 177 | 36 | 5.26 |
| 2HCZ | 245 | 123 | 25 | 5.26 |
| 1PBH | 317 | 192 | 39 | 5.26 |
| 1U67 | 600 | 384 | 78 | 5.26 |
| 2Z81 | 549 | 340 | 69 | 5.26 |
| 2G6D | 217 | 138 | 28 | 5.26 |
| 1IEW | 605 | 493 | 100 | 5.26 |
| 1IEQ | 605 | 479 | 97 | 5.26 |
| 1WBA | 175 | 89 | 18 | 5.26 |
| 1VCJ | 389 | 183 | 37 | 5.26 |
| 1TDO | 486 | 391 | 79 | 5.27 |
| 1J8V | 605 | 490 | 99 | 5.27 |
| 2ZJH | 405 | 203 | 41 | 5.27 |
| 1JZU | 157 | 109 | 22 | 5.27 |
| 2VIE | 392 | 223 | 45 | 5.27 |
| 1TZQ | 175 | 114 | 23 | 5.27 |
| 2O6X | 310 | 248 | 50 | 5.27 |
| 1BK1 | 184 | 124 | 25 | 5.27 |
| 1TIB | 269 | 184 | 37 | 5.27 |
| 1MZ6 | 638 | 383 | 77 | 5.27 |
| 154L | 185 | 194 | 39 | 5.27 |
| 1YT4 | 263 | 249 | 50 | 5.27 |
| 2H4R | 415 | 284 | 57 | 5.27 |
| 2JX9 | 106 | 55 | 11 | 5.27 |
| 1J1F | 191 | 140 | 28 | 5.27 |
| 1JWT | 305 | 160 | 32 | 5.27 |
| 3DHM | 100 | 50 | 10 | 5.27 |
| 1BOY | 219 | 105 | 21 | 5.27 |
| 1XYQ | 111 | 80 | 16 | 5.27 |
| 2DN6 | 115 | 65 | 13 | 5.27 |
| 1IGX | 576 | 390 | 78 | 5.27 |
| 2VIZ | 392 | 225 | 45 | 5.27 |
| 3BUH | 409 | 220 | 44 | 5.27 |
| 1U5X | 140 | 75 | 15 | 5.27 |
| 1CX1 | 153 | 70 | 14 | 5.27 |
| 1AKN | 579 | 385 | 77 | 5.27 |
| 2E2O | 299 | 310 | 62 | 5.27 |
| 1EQC | 394 | 321 | 64 | 5.27 |
| 2AEZ | 543 | 301 | 60 | 5.28 |
| 1FHL | 334 | 286 | 57 | 5.28 |
| 2VIJ | 392 | 226 | 45 | 5.28 |
| 2PQ6 | 482 | 407 | 81 | 5.28 |
| 1RNE | 340 | 201 | 40 | 5.28 |
| 1LLN | 262 | 166 | 33 | 5.28 |
| 1CVZ | 212 | 146 | 29 | 5.28 |
| 1RMG | 422 | 292 | 58 | 5.28 |
| 1IAD | 200 | 136 | 27 | 5.28 |
| 1XW2 | 405 | 378 | 75 | 5.28 |
| 1TEM | 263 | 247 | 49 | 5.28 |
| 2ZHT | 411 | 222 | 44 | 5.28 |
| 2ZHV | 411 | 222 | 44 | 5.28 |
| 2GJ5 | 162 | 106 | 21 | 5.28 |
| 3BUF | 409 | 212 | 42 | 5.28 |
| 1FYV | 161 | 106 | 21 | 5.28 |
| 1OGO | 574 | 318 | 63 | 5.28 |
| 2JSY | 167 | 101 | 20 | 5.28 |
| 2Q2M | 162 | 96 | 19 | 5.28 |
| 1OC5 | 364 | 283 | 56 | 5.28 |
| 1ZU8 | 361 | 273 | 54 | 5.28 |
| 1PLR | 258 | 182 | 36 | 5.28 |
| 2C6U | 122 | 86 | 17 | 5.28 |
| 1FQG | 263 | 258 | 51 | 5.28 |
| 1E66 | 543 | 415 | 82 | 5.28 |
| 1ROA | 122 | 81 | 16 | 5.28 |
| 2PF0 | 400 | 319 | 63 | 5.28 |
| 1CJ5 | 162 | 76 | 15 | 5.28 |
| 1FAO | 126 | 71 | 14 | 5.28 |
| 1T7X | 278 | 213 | 42 | 5.28 |
| 1XQO | 256 | 274 | 54 | 5.28 |
| 2RKQ | 169 | 137 | 27 | 5.28 |
| 1XOG | 387 | 198 | 39 | 5.28 |
| 1GBS | 185 | 183 | 36 | 5.28 |
| 2CVB | 188 | 122 | 24 | 5.28 |
| 1N6V | 212 | 117 | 23 | 5.29 |
| 1JQR | 174 | 117 | 23 | 5.29 |
| 1IEH | 135 | 56 | 11 | 5.29 |
| 2PBO | 400 | 321 | 63 | 5.29 |
| 2PC8 | 400 | 316 | 62 | 5.29 |
| 2EBF | 746 | 622 | 122 | 5.29 |
| 3CJ7 | 456 | 357 | 70 | 5.29 |
| 1ERO | 263 | 245 | 48 | 5.29 |
| 1H14 | 405 | 383 | 75 | 5.29 |
| 1G6E | 87 | 46 | 9 | 5.29 |
| 3DTM | 263 | 230 | 45 | 5.29 |
| 1T7Y | 278 | 220 | 43 | 5.29 |
| 1SYT | 361 | 287 | 56 | 5.29 |
| 1Z7C | 191 | 159 | 31 | 5.29 |
| 1ST8 | 543 | 303 | 59 | 5.29 |
| 1Z2E | 139 | 113 | 22 | 5.29 |
| 2PFC | 183 | 113 | 22 | 5.29 |
| 1UCD | 190 | 149 | 29 | 5.29 |
| 1YJL | 306 | 149 | 29 | 5.29 |
| 1FCQ | 350 | 283 | 55 | 5.29 |
| 2P3X | 339 | 211 | 41 | 5.29 |
| 1MR5 | 648 | 381 | 74 | 5.29 |
| 2B31 | 361 | 273 | 53 | 5.29 |
| 2ZDZ | 415 | 217 | 42 | 5.30 |
| 1T7W | 278 | 217 | 42 | 5.30 |
| 1LQ2 | 602 | 465 | 90 | 5.30 |
| 2NQ5 | 755 | 620 | 120 | 5.30 |
| 2R2J | 382 | 243 | 47 | 5.30 |
| 2K5O | 114 | 88 | 17 | 5.30 |
| 2EJT | 378 | 316 | 61 | 5.30 |
| 2HM1 | 406 | 228 | 44 | 5.30 |
| 2Q8W | 261 | 223 | 43 | 5.30 |
| 1PNG | 314 | 166 | 32 | 5.30 |
| 1RPA | 342 | 275 | 53 | 5.30 |
| 1N6U | 212 | 109 | 21 | 5.30 |
| 2CKM | 543 | 405 | 78 | 5.30 |
| 1JNE | 420 | 322 | 62 | 5.30 |
| 1RW5 | 199 | 187 | 36 | 5.30 |
| 1VXO | 537 | 400 | 77 | 5.30 |
| 3DHJ | 100 | 52 | 10 | 5.30 |
| 3C9M | 348 | 286 | 55 | 5.30 |
| 1YNA | 194 | 130 | 25 | 5.30 |
| 1QO9 | 585 | 385 | 74 | 5.30 |
| 1OG3 | 226 | 177 | 34 | 5.30 |
| 1CZ1 | 394 | 328 | 63 | 5.30 |
| 1IV8 | 720 | 547 | 105 | 5.30 |
| 1BSO | 162 | 99 | 19 | 5.30 |
| 1Z3R | 99 | 47 | 9 | 5.30 |
| 1ACZ | 108 | 47 | 9 | 5.30 |
| 1FE2 | 576 | 397 | 76 | 5.30 |
| 1JWZ | 263 | 251 | 48 | 5.30 |
| 1ERQ | 263 | 251 | 48 | 5.30 |
| 1VXR | 537 | 408 | 78 | 5.30 |
| 1WCU | 153 | 89 | 17 | 5.31 |
| 1H13 | 405 | 393 | 75 | 5.31 |
| 1E9L | 377 | 283 | 54 | 5.31 |
| 1GIK | 261 | 194 | 37 | 5.31 |
| 1ACJ | 537 | 404 | 77 | 5.31 |
| 2ODQ | 509 | 336 | 64 | 5.31 |
| 1ERM | 263 | 252 | 48 | 5.31 |
| 1MU0 | 293 | 252 | 48 | 5.31 |
| 1XWT | 405 | 378 | 72 | 5.31 |
| 2CVS | 888 | 552 | 105 | 5.31 |
| 1LSP | 185 | 163 | 31 | 5.31 |
| 2DT3 | 361 | 284 | 54 | 5.31 |
| 3TGL | 269 | 221 | 42 | 5.31 |
| 2QU2 | 415 | 221 | 42 | 5.31 |
| 1T7V | 278 | 221 | 42 | 5.31 |
| 2Q99 | 381 | 279 | 53 | 5.31 |
| 1GY0 | 226 | 179 | 34 | 5.31 |
| 1WCS | 641 | 358 | 68 | 5.31 |
| 2ZHU | 411 | 216 | 41 | 5.31 |
| 1FCV | 350 | 269 | 51 | 5.31 |
| 1GX8 | 162 | 111 | 21 | 5.31 |
| 1IGZ | 576 | 402 | 76 | 5.31 |
| 1PGS | 314 | 164 | 31 | 5.31 |
| 1UCT | 218 | 90 | 17 | 5.31 |
| 2B4F | 405 | 376 | 71 | 5.31 |
| 1ZG4 | 286 | 249 | 47 | 5.31 |
| 2ADD | 543 | 302 | 57 | 5.31 |
| 2DSV | 361 | 281 | 53 | 5.31 |
| 2DSZ | 361 | 281 | 53 | 5.31 |
| 1Q35 | 320 | 281 | 53 | 5.31 |
| 1GPN | 537 | 414 | 78 | 5.31 |
| 2DT1 | 361 | 292 | 55 | 5.32 |
| 1LN6 | 348 | 186 | 35 | 5.32 |
| 2Z1S | 454 | 372 | 70 | 5.32 |
| 2QRL | 394 | 287 | 54 | 5.32 |
| 1T80 | 278 | 218 | 41 | 5.32 |
| 1H23 | 543 | 415 | 78 | 5.32 |
| 2G41 | 361 | 277 | 52 | 5.32 |
| 2JYP | 36 | 16 | 3 | 5.32 |
| 1BMG | 98 | 48 | 9 | 5.32 |
| 1AG2 | 103 | 80 | 15 | 5.32 |
| 2ICC | 119 | 64 | 12 | 5.32 |
| 2Q1M | 130 | 64 | 12 | 5.32 |
| 1PKO | 139 | 64 | 12 | 5.32 |
| 1L6Z | 216 | 96 | 18 | 5.32 |
| 2OXB | 537 | 272 | 51 | 5.32 |
| 1VF8 | 377 | 315 | 59 | 5.32 |
| 2ADE | 543 | 305 | 57 | 5.32 |
| 2DSW | 361 | 289 | 54 | 5.32 |
| 1EEA | 534 | 380 | 71 | 5.32 |
| 1XRV | 361 | 295 | 55 | 5.32 |
| 1BSY | 162 | 102 | 19 | 5.32 |
| 1XH0 | 496 | 349 | 65 | 5.32 |
| 2INT | 129 | 129 | 24 | 5.32 |
| 1WRJ | 156 | 129 | 24 | 5.32 |
| 2QQJ | 325 | 156 | 29 | 5.32 |
| 2EJU | 378 | 323 | 60 | 5.32 |
| 1TV2 | 458 | 431 | 80 | 5.33 |
| 1LF4 | 331 | 194 | 36 | 5.33 |
| 1IEV | 605 | 464 | 86 | 5.33 |
| 2DT0 | 361 | 286 | 53 | 5.33 |
| 2JXA | 106 | 54 | 10 | 5.33 |
| 1AC0 | 108 | 54 | 10 | 5.33 |
| 2FY7 | 287 | 189 | 35 | 5.33 |
| 1XWQ | 405 | 378 | 70 | 5.33 |
| 2G5X | 234 | 200 | 37 | 5.33 |
| 1ZG6 | 286 | 238 | 44 | 5.33 |
| 3D2H | 538 | 395 | 73 | 5.33 |
| 1IJB | 202 | 184 | 34 | 5.33 |
| 2V5M | 388 | 195 | 36 | 5.33 |
| 1CFJ | 537 | 401 | 74 | 5.33 |
| 1TFV | 361 | 271 | 50 | 5.33 |
| 1NGL | 179 | 103 | 19 | 5.33 |
| 2O9O | 361 | 282 | 52 | 5.33 |
| 1VOT | 537 | 396 | 73 | 5.33 |
| 2PI6 | 361 | 293 | 54 | 5.33 |
| 1HJD | 101 | 38 | 7 | 5.33 |
| 1BT3 | 345 | 228 | 42 | 5.33 |
| 2R4V | 247 | 190 | 35 | 5.33 |
| 1TV3 | 458 | 429 | 79 | 5.33 |
| 2CMF | 543 | 391 | 72 | 5.33 |
| 1IXK | 315 | 239 | 44 | 5.33 |
| 1JND | 420 | 326 | 60 | 5.33 |
| 1ZGB | 543 | 413 | 76 | 5.33 |
| 2OYU | 600 | 424 | 78 | 5.33 |
| 1XYH | 161 | 98 | 18 | 5.33 |
| 2DPE | 361 | 294 | 54 | 5.33 |
| 2SIL | 381 | 218 | 40 | 5.33 |
| 2CEK | 535 | 409 | 75 | 5.33 |
| 3BJ9 | 116 | 60 | 11 | 5.33 |
| 2A8Z | 405 | 360 | 66 | 5.33 |
| 1XRN | 293 | 251 | 46 | 5.33 |
| 1K4Y | 534 | 382 | 70 | 5.33 |
| 1I5P | 633 | 513 | 94 | 5.33 |
| 1NPU | 117 | 71 | 13 | 5.33 |
| 3CSP | 139 | 71 | 13 | 5.33 |
| 2DT2 | 361 | 284 | 52 | 5.33 |
| 1LJY | 361 | 284 | 52 | 5.33 |
| 2JIE | 454 | 388 | 71 | 5.33 |
| 3BCI | 186 | 164 | 30 | 5.33 |
| 1WOU | 123 | 93 | 17 | 5.34 |
| 1MT3 | 293 | 230 | 42 | 5.34 |
| 2C5F | 537 | 406 | 74 | 5.34 |
| 1T7Z | 278 | 214 | 39 | 5.34 |
| 2QF8 | 361 | 280 | 51 | 5.34 |
| 2ESC | 361 | 291 | 53 | 5.34 |
| 1H22 | 543 | 412 | 75 | 5.34 |
| 2P39 | 155 | 66 | 12 | 5.34 |
| 1EW3 | 159 | 110 | 20 | 5.34 |
| 2O92 | 361 | 275 | 50 | 5.34 |
| 1BU8 | 452 | 303 | 55 | 5.34 |
| 1FO9 | 348 | 248 | 45 | 5.34 |
| 1C2B | 540 | 397 | 72 | 5.34 |
| 1EVE | 543 | 386 | 70 | 5.34 |
| 1ARL | 307 | 254 | 46 | 5.34 |
| 1ISR | 490 | 326 | 59 | 5.34 |
| 1KDK | 177 | 94 | 17 | 5.34 |
| 2O9R | 452 | 393 | 71 | 5.34 |
| 3CIG | 697 | 377 | 68 | 5.34 |
| 1GPK | 537 | 416 | 75 | 5.34 |
| 2AC1 | 541 | 294 | 53 | 5.34 |
| 1NPR | 248 | 172 | 31 | 5.34 |
| 2VB5 | 100 | 50 | 9 | 5.35 |
| 2D3V | 196 | 89 | 16 | 5.35 |
| 3CRW | 551 | 356 | 64 | 5.35 |
| 1JJB | 532 | 401 | 72 | 5.35 |
| 1QHT | 775 | 575 | 103 | 5.35 |
| 3D2D | 538 | 380 | 68 | 5.35 |
| 2BAG | 543 | 403 | 72 | 5.35 |
| 1KOE | 172 | 112 | 20 | 5.35 |
| 1EA5 | 537 | 409 | 73 | 5.35 |
| 3BLG | 162 | 101 | 18 | 5.35 |
| 2Q98 | 191 | 202 | 36 | 5.35 |
| 1NST | 325 | 236 | 42 | 5.35 |
| 2JG0 | 535 | 472 | 84 | 5.35 |
| 2GTO | 96 | 45 | 8 | 5.35 |
| 1GQR | 532 | 405 | 72 | 5.35 |
| 1S0J | 648 | 377 | 67 | 5.35 |
| 1S3A | 102 | 62 | 11 | 5.35 |
| 2ACE | 537 | 389 | 69 | 5.35 |
| 2QU3 | 415 | 226 | 40 | 5.36 |
| 2GGO | 401 | 277 | 49 | 5.36 |
| 1LEE | 331 | 198 | 35 | 5.36 |
| 1OCE | 537 | 368 | 65 | 5.36 |
| 4TGL | 269 | 204 | 36 | 5.36 |
| 2JOM | 148 | 85 | 15 | 5.36 |
| 3SEB | 238 | 153 | 27 | 5.36 |
| 1QTI | 537 | 397 | 70 | 5.36 |
| 1VZ3 | 710 | 471 | 83 | 5.36 |
| 2RIP | 729 | 454 | 80 | 5.36 |
| 2B5M | 1140 | 591 | 104 | 5.36 |
| 1HBJ | 543 | 415 | 73 | 5.36 |
| 1WOL | 122 | 148 | 26 | 5.36 |
| 2B7U | 257 | 205 | 36 | 5.36 |
| 1UT6 | 537 | 416 | 73 | 5.36 |
| 1DIY | 553 | 399 | 70 | 5.36 |
| 1GAK | 141 | 171 | 30 | 5.36 |
| 1GKU | 1054 | 845 | 148 | 5.36 |
| 1OP4 | 159 | 40 | 7 | 5.36 |
| 1XRQ | 293 | 263 | 46 | 5.36 |
| 2I35 | 348 | 309 | 54 | 5.36 |
| 1SR0 | 361 | 269 | 47 | 5.36 |
| 1MTZ | 293 | 269 | 47 | 5.36 |
| 1LF3 | 331 | 189 | 33 | 5.37 |
| 1EBV | 551 | 424 | 74 | 5.37 |
| 2IGX | 329 | 195 | 34 | 5.37 |
| 1OG4 | 226 | 178 | 31 | 5.37 |
| 1OG1 | 226 | 184 | 32 | 5.37 |
| 1F6W | 533 | 369 | 64 | 5.37 |
| 1RKM | 517 | 398 | 69 | 5.37 |
| 1XQV | 293 | 254 | 44 | 5.37 |
| 1ITF | 165 | 168 | 29 | 5.37 |
| 2BCE | 579 | 400 | 69 | 5.37 |
| 1W4L | 543 | 412 | 71 | 5.37 |
| 1VZ2 | 710 | 471 | 81 | 5.37 |
| 1ODC | 543 | 413 | 71 | 5.37 |
| 1B0O | 162 | 99 | 17 | 5.38 |
| 1Q56 | 195 | 99 | 17 | 5.38 |
| 1GQS | 532 | 379 | 65 | 5.38 |
| 2FJ0 | 551 | 392 | 67 | 5.38 |
| 1HJQ | 332 | 281 | 48 | 5.38 |
| 1KEX | 155 | 82 | 14 | 5.38 |
| 1SAU | 115 | 100 | 17 | 5.38 |
| 1S0I | 648 | 377 | 64 | 5.38 |
| 1XHG | 361 | 278 | 47 | 5.39 |
| 1FWV | 134 | 71 | 12 | 5.39 |
| 1XRR | 293 | 255 | 43 | 5.39 |
| 2C9K | 612 | 447 | 75 | 5.39 |
| 3BD2 | 186 | 167 | 28 | 5.39 |
| 2DFP | 534 | 406 | 68 | 5.39 |
| 3BD9 | 280 | 209 | 35 | 5.39 |
| 1DX6 | 543 | 407 | 68 | 5.39 |
| 2HGF | 97 | 54 | 9 | 5.39 |
| 1Z66 | 96 | 54 | 9 | 5.39 |
| 1E5T | 710 | 480 | 80 | 5.39 |
| 1PJA | 302 | 223 | 37 | 5.40 |
| 2O9P | 454 | 398 | 66 | 5.40 |
| 1RLR | 761 | 585 | 97 | 5.40 |
| 3BCK | 186 | 157 | 26 | 5.40 |
| 2O9T | 454 | 393 | 65 | 5.40 |
| 1JFP | 348 | 200 | 33 | 5.40 |
| 2DRU | 180 | 97 | 16 | 5.40 |
| 1OWQ | 361 | 291 | 48 | 5.40 |
| 1MF7 | 194 | 170 | 28 | 5.40 |
| 2ORX | 314 | 152 | 25 | 5.40 |
| 1SE4 | 239 | 146 | 24 | 5.40 |
| 1XRO | 293 | 262 | 43 | 5.40 |
| 1TGO | 773 | 640 | 105 | 5.40 |
| 1QQF | 277 | 293 | 48 | 5.40 |
| 1V03 | 565 | 434 | 71 | 5.41 |
| 1XQW | 293 | 263 | 43 | 5.41 |
| 1BSQ | 162 | 98 | 16 | 5.41 |
| 2YXF | 100 | 49 | 8 | 5.41 |
| 2OQP | 134 | 80 | 13 | 5.41 |
| 1LF2 | 331 | 197 | 32 | 5.41 |
| 1GCF | 109 | 37 | 6 | 5.41 |
| 1SE3 | 239 | 148 | 24 | 5.41 |
| 1RSF | 126 | 68 | 11 | 5.41 |
| 1E3G | 263 | 272 | 44 | 5.41 |
| 1XRL | 293 | 266 | 43 | 5.41 |
| 1GX9 | 162 | 99 | 16 | 5.41 |
| 1KKH | 317 | 285 | 46 | 5.41 |
| 2JOH | 148 | 93 | 15 | 5.41 |
| 1FWU | 134 | 69 | 11 | 5.42 |
| 1DQO | 135 | 69 | 11 | 5.42 |
| 2BJU | 453 | 201 | 32 | 5.42 |
| 1MQA | 180 | 126 | 20 | 5.42 |
| 1UU6 | 224 | 145 | 23 | 5.42 |
| 1S0G | 1290 | 972 | 154 | 5.42 |
| 1EH9 | 558 | 343 | 54 | 5.43 |
| 1XRM | 293 | 261 | 41 | 5.43 |
| 1VHP | 117 | 51 | 8 | 5.43 |
| 2W2S | 202 | 115 | 18 | 5.43 |
| 1OLR | 224 | 147 | 23 | 5.43 |
| 3CTK | 248 | 211 | 33 | 5.43 |
| 2R30 | 130 | 64 | 10 | 5.43 |
| 1CCZ | 171 | 90 | 14 | 5.43 |
| 1QGV | 142 | 103 | 16 | 5.44 |
| 2QQI | 318 | 149 | 23 | 5.44 |
| 1GYD | 315 | 175 | 27 | 5.44 |
| 2EHG | 149 | 143 | 22 | 5.44 |
| 2QLY | 870 | 586 | 90 | 5.44 |
| 1ZRH | 274 | 203 | 31 | 5.44 |
| 1XQX | 293 | 256 | 39 | 5.45 |
| 4GCR | 174 | 92 | 14 | 5.45 |
| 1C3D | 294 | 309 | 47 | 5.45 |
| 1EHA | 558 | 350 | 53 | 5.45 |
| 1CZS | 160 | 86 | 13 | 5.45 |
| 1CZT | 160 | 86 | 13 | 5.45 |
| 1SNT | 382 | 193 | 29 | 5.45 |
| 1GXA | 162 | 100 | 15 | 5.45 |
| 1UU4 | 224 | 147 | 22 | 5.46 |
| 1Z2K | 109 | 47 | 7 | 5.46 |
| 1POQ | 118 | 54 | 8 | 5.46 |
| 1J8S | 196 | 95 | 14 | 5.46 |
| 2ECE | 462 | 246 | 36 | 5.47 |
| 2RM5 | 167 | 103 | 15 | 5.47 |
| 1SE2 | 239 | 133 | 19 | 5.48 |
| 1N9D | 199 | 172 | 24 | 5.49 |
| 1J8R | 196 | 97 | 13 | 5.51 |
| 1P6F | 242 | 98 | 13 | 5.52 |
| 2YX0 | 342 | 267 | 35 | 5.52 |
| 3BN6 | 158 | 85 | 11 | 5.53 |
| 1PFC | 113 | 31 | 4 | 5.53 |
| 1F53 | 84 | 48 | 6 | 5.55 |
| 2F09 | 102 | 40 | 5 | 5.55 |
| 1OLL | 188 | 98 | 12 | 5.55 |
